# Low and high carbohydrate isocaloric diets on performance, fat oxidation, glucose and cardiometabolic health in middle age males

**DOI:** 10.3389/fnut.2023.1084021

**Published:** 2023-02-09

**Authors:** Philip J. Prins, Timothy D. Noakes, Alex Buga, Dominic P. D’Agostino, Jeff S. Volek, Jeffrey D. Buxton, Kara Heckman, Dalton W. Jones, Naomi E. Tobias, Holly M. Grose, Anna K. Jenkins, Kelli T. Jancay, Andrew P. Koutnik

**Affiliations:** ^1^Department of Exercise Science, Grove City College, Grove City, PA, United States; ^2^Department of Medical and Wellness Science, Cape Peninsula University of Technology, Cape Town, South Africa; ^3^Department of Human Sciences, The Ohio State University, Columbus, OH, United States; ^4^Department of Molecular Pharmacology and Physiology, University of South Florida, Tampa, FL, United States; ^5^Nebraska Methodist Health System, Omaha, NE, United States; ^6^Human Healthspan, Resilience, and Performance, Institute for Human and Machine Cognition, Pensacola, FL, United States

**Keywords:** high fat diet, low-carbohydrate, high-carbohydrate, fat oxidation, carbohydrate oxidation, prediabetes

## Abstract

High carbohydrate, low fat (HCLF) diets have been the predominant nutrition strategy for athletic performance, but recent evidence following multi-week habituation has challenged the superiority of HCLF over low carbohydrate, high fat (LCHF) diets, along with growing interest in the potential health and disease implications of dietary choice. Highly trained competitive middle-aged athletes underwent two 31-day isocaloric diets (HCLF or LCHF) in a randomized, counterbalanced, and crossover design while controlling calories and training load. Performance, body composition, substrate oxidation, cardiometabolic, and 31-day minute-by-minute glucose (CGM) biomarkers were assessed. We demonstrated: (i) equivalent high-intensity performance (@∼85%VO_2max_), fasting insulin, hsCRP, and HbA_1c_ without significant body composition changes across groups; (ii) record high peak fat oxidation rates (LCHF:1.58 ± 0.33g/min @ 86.40 ± 6.24%VO_2max_; 30% subjects > 1.85 g/min); (iii) higher total, LDL, and HDL cholesterol on LCHF; (iv) reduced glucose mean/median and variability on LCHF. We also found that the 31-day mean glucose on HCLF predicted 31-day glucose reductions on LCHF, and the 31-day glucose reduction on LCHF predicted LCHF peak fat oxidation rates. Interestingly, 30% of athletes had 31-day mean, median and fasting glucose > 100 mg/dL on HCLF (range: 111.68-115.19 mg/dL; consistent with pre-diabetes), also had the largest glycemic and fat oxidation response to carbohydrate restriction. These results: (i) challenge whether higher carbohydrate intake is superior for athletic performance, even during shorter-duration, higher-intensity exercise; (ii) demonstrate that lower carbohydrate intake may be a therapeutic strategy to independently improve glycemic control, particularly in those at risk for diabetes; (iii) demonstrate a unique relationship between continuous glycemic parameters and systemic metabolism.

## Introduction

From 1896 to 2008, athletes competing in the Olympics demonstrated trends for increased carbohydrate intake in 1976 and a predominant shift toward high-carbohydrate low-fat (HCLF) diets in the 1996 Olympic games (1–3). This shift in athlete food preference toward carbohydrates was cited to be driven by (i) increased user consciousness of healthy food choice to optimized performance (1, 4, 5); (ii) the importance of muscle glycogen as the preferable metabolic fuel during exercise of either high intensity or long duration low intensity (6–12) following the emergence of muscle biopsy techniques in 1960s (13); (iii) anaplerotic theory in which depleted muscle glycogen attenuates mitochondrial oxaloacetate concentrations and thus reduced mitochondrial capacity to oxidize fatty acids (14); (iv) multiple studies illustrating carbohydrate ingestion delayed or reversed fatigue by maintaining blood glucose homeostasis (15–17); (v) “cross-over effect” (18–20) in which exercise of increasing intensity becomes increasingly dependent on carbohydrate oxidation since fat oxidation effectively ceases at any exercise intensity ≥ 85% VO_2max_ (18–22); (vi) clinical trials of high-fat diets resulting in impaired performance in both recreational (23) and elite athletes (i.e., Olympic class) (24–26).

Countering this evidence is a growing body of data demonstrating that extended habituation to a low-carbohydrate high-fat (LCHF) diet can shift the “cross-over” set-point in favor of greater fat oxidation, even at much higher intensities [(< 85% VO_2max_); (27, 28)], and dramatically increase the rates of peak fat oxidation at moderate intensities (i.e., 60% VO_2max_) (29). Rates of fat oxidation during exercise across these LCHF studies are amongst the highest yet measured (24, 25, 28, 30–32) even though they were measured during progressive exercise to exhaustion (e.g., minutes), rather than more prolonged exercise (e.g., hours). These studies opened key questions of whether performance-equivalence would still hold (i) if exercise intensity was increased (i.e., running trial 800-1,609 m) and (ii) if high-intensity interval sessions would facilitate more muscle glycogen depletion in those habituated to LCHF which would become more apparent as the number of intervals increased.

**FIGURE 1:**
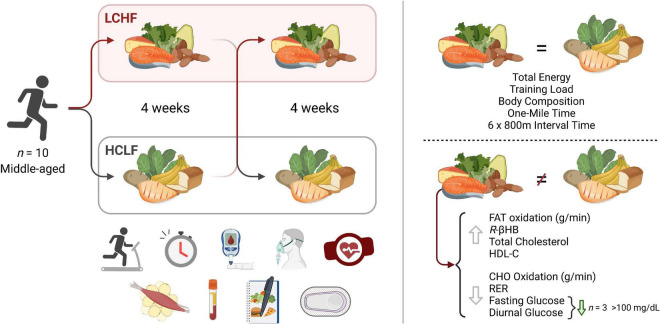
Low and high carbohydrate isocaloric diets on performance, fat oxidation, glucose and cardiometabolic health in middle age males. Highly trained competitive middle-aged male athletes underwent two 31-day isocaloric diets (HCLF or LCHF) in a randomized, counterbalanced, and crossover design while controlling calories and training load. Performance, body composition, substrate oxidation, cardiometabolic, and 31-day minute-by-minute glucose (CGM) biomarkers were assessed. We demonstrated: (i) equivalent high-intensity performance (@∼85%VO2max), fasting insulin, hsCRP, and HbA1c without significant body composition changes across groups; (ii) record high peak fat oxidation rates (LCHF:1.58 ± 0.33g/min) at 86.40 ± 6.24%VO2max with 30% subjects > 1.85g/min; (iii) higher total, LDL, and HDL cholesterol on LCHF; (iv) reduced glucose mean, median, and variability on LCHF. We also found that the 31-day mean glucose on HCLF predicted 31-day glucose reductions on LCHF, and the 31-day glucose reduction on LCHF predicted LCHF peak fat oxidation rates. 30% of athletes had 31-day mean, median and fasting glucose > 100mg/dL on HCLF (range:111.68-115.19 mg/dL; consistent with pre-diabetes), also had the largest glycemic and fat oxidation response to carbohydrate restriction. CHO, Carbohydrate; HCLF, High-Carbohydrate Low-Fat Diet; HDL-C, High Density Lipoprotein Cholesterol; LCHF, Low-Carbohydrate High-Fat Diet; *R*-βHB, *R*-β-Hydroxybutyrate; RER, Respiratory Exchange Ratio.

Paramount to both athletes and the general population is the potential health and disease implication of their dietary choice, particularly as individuals advance in age. Multiple studies have illustrated beneficial shifts in glucose, insulin, hemoglobin A_1c_ (HbA_1c_), inflammation, and oxidative stress biomarkers, along with proposed alterations in diabetes, cancer, neurological, and other disease clinical outcomes resulting from LCHF diets and their resulting metabolite shifts (33–40). While there is evidence for optimism, some dating back over a century (36, 38), there has been a call for more high-quality evidence (41). Thus, leveraging a randomized within-subject controlled cross-over design in middle-aged competitive athletes, we sought to rigorously address these gaps and evaluate the effects of 4-week habituation to either an LCHF or HCLF diet while controlling calories and training volume across groups on (i) high-intensity short duration exercise performance, (ii) body composition, (iii) metabolite oxidation rates at graded exercise intensities, and the (iv) continuous glycemic, insulin and overall cardiometabolic biomarker changes which accompanied these multiweek dietary patterns. We hypothesized that LCHF habituation would result in reduced high-intensity exercise performance. Interestingly, without changes in calories or training load, or significant changes in body composition across groups, the results found herein challenge the current superiority of high-carbohydrate diets (even during high-intensity exercise) for performance, illustrate record high rates of fat oxidation, demonstrate unique and consistent changes in continuous glucose which predict systemic fat oxidation rates, and a surprising incidence of glycemic values consistent with pre-diabetes phenotype in this middle-aged competitive athlete cohort during high carbohydrate consumption which was therapeutically addressed with carbohydrate restriction (Figure 1).

## Materials and methods

### Experimental design

**FIGURE 2:**
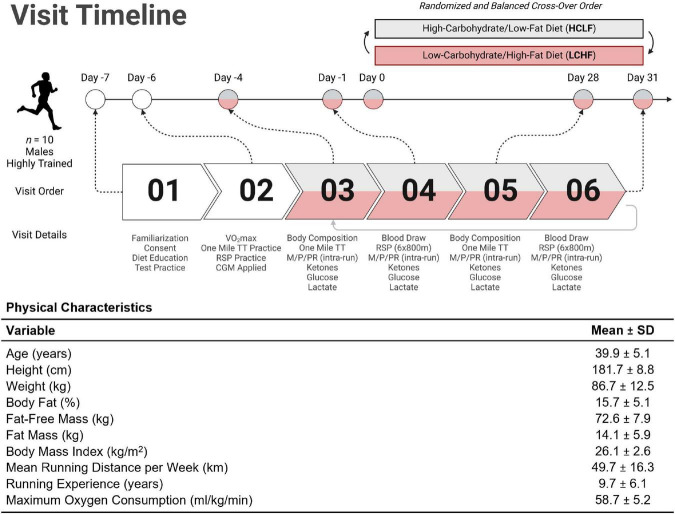
Experimental timeline and participant characteristics. Highly trained middle-aged male runners (*n* = 10) underwent a battery of tests throughout the study. The Pre-diet study tasks included familiarization with the study protocol, time trial (TT) practice runs, VO2max assessment, and continuous glucose monitor (CGM) application for measurement of interstitial fluid glucose (visits 01 and 02). Ketones (R-β-hydroxybutyrate) and lactate were analyzed enzymatically using finger-sticks. All participants were assessed for body composition changes; metabolic, physiological, and perceptual assessments (M/P/PR); venous blood draws; ketone/glucose/lactate measurements; one mile TT; and repeat sprint protocol (RSP; 6 × 800m) from visit 03 through 06. Diet order was randomized and balanced to explore the between/within-participant cross-over effects. Once a first diet was completed, subjects crossed over to the other dietary treatment and repeated visits 03 through 06 (i.e., visits 07 through 10). The table illustrates the mean (SD) participant age, anthropometry, and training status variables collected at visit 01. TT = time trial; RSP = repeated sprint protocol; LCHF = low-carbohydrate/high-fat diet; HCLF = high-carbohydrate/low-fat diet; M/P/PR = metabolic, physiological, and perceptual. Figure was created with BioRender.com.

Highly trained competitive middle-aged athletes underwent two 31-day isocaloric diet periods (HCLF or LCHF) in a randomized (www.randomizer.org), counterbalanced, crossover design with a two-week washout period between dietary interventions without feeding limitations. We assessed both diets while controlling calories and training load. Primary outcomes were performance, substrate oxidation during exercise, continuous glucose and cardiometabolic biomarkers. Each subject visited the laboratory on ten separate occasions, performing testing before (PRE) and at the completion (POST) of each 31-day dietary intervention (Figure 2). Visit one and two consisted of a familiarization of measurement instruments, equipment, perceptual measurements (42, 43), consent, VO_2_max test (31), and continuous glucose monitoring (CGM; Freestyle Libre 2, Abbott Diabetes Care Inc; Almeda, CA) sensor application. One-mile [1,609 m] running time trial (TT) and repeated sprint protocol (RSP; 6 × 800 m) were performed twice on each dietary intervention (Pre and Post). One-mile TT was performed on Day –4 and Day 28. Three days later, subjects performed the RSP on Day –1 and Day 31. Gas exchange and perceptual changes were recorded throughout each performance trial Pre and Post each dietary intervention. Body composition and cardiometabolic parameters were measured Pre and Post each dietary intervention. Capillary and interstitial metabolite concentrations were measured throughout each 31-day dietary intervention period. Testing sessions were conducted at the same time of day in an environment with controlled temperature and humidity (19-21 ^0^C, humidity = 35-40%) within the Exercise Science Laboratory of Grove City College. The experimental protocol was approved by the Institutional Review Board of Grove City College prior to implementation (IRB number 110-2021).

### Subjects

Ten middle-aged competitive distance runners (Figure 2) were recruited directly from local running organizations and by advertising within the local community. Inclusion and exclusion criteria were screened utilizing medical history/Physical Activity Readiness Questionnaire (PAR-Q). Inclusion criteria included: (1) 30 to 50-years old males; (2) completion of one-mile (1,609 meter) run in under seven minutes; (3) running ≥ 20 miles (32 kilometers) per week; (4) > 2 years of running experience; (5) currently consuming a carbohydrate-based diet (> 50% kcals). Exclusion Criteria included: (1) cigarette smoker; (2) metabolic or cardiovascular disease, (3) orthopedic, musculoskeletal, neurological, and/or any medical conditions that prohibit exercise; (4) psychiatric disorder; (5) pharmaceutical drugs affecting measurement parameters. Subjects were prohibited from using any ergogenic aids/supplements one month prior to study initiation to study completion. Subjects were instructed to refrain from caffeine and alcohol consumption for 48 h, and racing or training for 24 h before each performance test.

### Dietary interventions

Using direct counseling and prepared educational handouts, a registered dietitian taught and guided each athlete prior to the experimental phase on how to implement the LCHF and HCLF diets at home. Instructional handouts included: (i) summary of key aspects of each diet, (ii) 31-day LCHF and HCLF meal plan but were advised to use this meal plan as a guide rather than a strict protocol; (iii) detailed guide on acceptable low-carbohydrate foods as well as a recommended list of nutritious fat and protein-rich foods. The primary macronutrient targets for LCHF and HCLF were expressed as both a percentage of total daily energy intake and daily gram intake: LCHF: < 50 g/day carbohydrate, 75–80% fat, 15–20% protein; HCLF: 60–65% carbohydrate, 20% fat, 15–20% protein (Table 1). Additionally, the LCHF group was recommended to supplement their diets with added salt to taste at mealtime and supplement 1-2 g/day of sodium from bouillon cubes, or homemade broth (44).

**TABLE 1:** Dietary composition.

Variable	LCHF	HCLF	*P*-value
Energy (kcal/day)	2545.3 ± 503.6	2595.7 ± 443.1	0.785
Carbohydrate (g)	40.9 ± 8.7	384.7 ± 65.3	<0.001
Protein (g)	141.0 ± 26.0	110.6 ± 20.8	0.010
Fat (g)	198.2 ± 45.2	67.6 ± 17.6	<0.001
Carbohydrate (%)	7.98 ± 3.49	63.2 ± 3.88	<0.001
Protein (%)	26.7 ± 7.32	17.7 ± 2.61	0.001
Fat (%)	64.4 ± 9.33	18.4 ± 6.12	<0.001
Cholesterol (mg)	839.7 ± 193.8	180.9 ± 66.2	<0.001
Fiber (g)	13.7 ± 6.51	31.2 ± 5.71	<0.001
Sugar (g)	13.1 ± 4.09	129.1 ± 37.1	<0.001

Participant dietary composition on low-carbohydrate high-fat (LCHF) and high-carbohydrate low-fat (HCLF) diets were captured using a 3-day food records including 1 weekend day using MyFitnessPal. Paired t-tests were conducted on 31-day averages. Every comparison was significant between-diet difference for energy intake. Every other variable demonstrated significant differences. *n* = 10. Values are Mean ± SD.

Weekly energy intake and relative macronutrient distribution were monitored and estimated via 3-day weighed food records, capturing two consecutive weekdays and a weekend day via the online smartphone application, MyFitnessPal. Subjects used digital kitchen scales to measure the weights of food portions for total energy intake estimates. Researchers administrated subject’s MyFitnessPal user accounts and therefore had the ability to assess and modify the subject’s macronutrient and micronutrient intake throughout the intervention (45). A two-week recovery macrocycle was incorporated between each dietary intervention without dietary restriction.

Verification of compliance to the LCHF diet was done via capillary blood ketone concentrations on days 3, 7, 14, 21, and 28 before ingesting breakfast and exercising. Capillary blood ketone concentrations (*R*-β-hydroxybutyrate; Precision Xtra, Abbott Diabetes Care Inc., Almeda, CA) was also measured immediately before and after the LCHF diet by the primary researcher. The results from the dietary intake and ketone measurements enabled the registered dietician to individually fine-tune participants’ diets, if necessary, via phone or email, thus ensuring continuous nutritional ketosis while on the LCHF diet.

### Physical activity monitoring

Participants were instructed to maintain a training log (mode, duration, and intensity of each workout) during the study intervention without increasing or decreasing the training load (46). Training load was calculated by using the session-RPE method (RPE x session duration [min]) (47).

### Body mass and body composition monitoring

Participants reported to the laboratory at least ≥ 3 h post-prandial, refraining from exercise for 48 h. The measurements of body mass were performed on a medical scale with a precision of 0.1 kg. Body composition was evaluated using the electrical impedance technique (Tanita^®^ MC-980U, Tanita Corporation, Inc., Tokyo, Japan).

### Maximal exercise testing

On the second visit, subjects performed an incremental test to exhaustion on a motorized treadmill (Trackmaster TMX425C treadmill, Newton, KS) utilizing the modified Astrand treadmill protocol. The treadmill was calibrated before each exercise test according to the manufacturer’s instructions. Oxygen consumption (VO_2_) and carbon dioxide production (VCO_2_) was assessed using an automated metabolic analyzer system (TrueOne 2400, ParvoMedics, Sandy, UT) calibrated prior to each exercise test using standard calibration gases (16% O_2_ and 4% CO_2_). Subjects wore a Polar heart rate monitor (Polar Electro, Kempele, Finland) during exercise to measure heart rate. After a thorough explanation of the experimental procedures, each subject was instructed to walk on the treadmill for 3 min as a warm-up at a self-selected speed (0% grade). Immediately following the 3-min warm-up, the speed was increased to 8–13 km.hr^–1^ for 3 min (0% grade) to achieve the subjects’ comfortable running pace. After 3 min of running at 0% grade, the grade was increased 2.5% every 2 min throughout the test protocol while speed was kept constant. At the end of the test, the highest VO_2_ value recorded during the last 30 seconds of exercise was considered the subject’s VO_2max_ (48). Some or all of the following criteria were used to determine if a physiologically valid VO_2max_ had been attained: (a) a plateau in VO_2_ with increasing exercise intensity (< 150 ml/min or < 2.1 ml/kg/min), (b) a respiratory exchange ratio (RER) of ≥ 1.1, (c) and volitional termination due to exhaustion.

### One-mile time trial

Participants arrived at the laboratory in the morning at least ≥ 3 h post-prandial and performed a one-mile TT on a motorized treadmill (TMX425C treadmill, Trackmaster, Newton, KS, USA). Subjects performed a warmup run consisting of 5 min at 45% VO_2max_ followed by 5 min at 65% VO_2max_ (10 min total). After a 5-min passive rest period, subjects then initiated the one-mile TT (1,609 m). Prior to the TT, the treadmill was brought to a standstill (0 km.hr^–1^), all timing devices were reset, the distance covered on the treadmill monitor was reset, a 5-s count down was given, and the TT began. Each TT commenced with a 30-second rolling start at a running speed approximating 80% VO_2max_, after which subjects could freely adjust their speed. Running speed and time was not visible to subjects during the TT. Subjects were verbally informed of the distance they had covered at 200m intervals. The treadmill gradient was set to 1% throughout the exercise (49). Subjects were instructed to finish the TT as fast as possible and were not informed of the overall performance time until completion of the final testing session. Heart rate (Polar Electro, Kempele, Finland), RPE (RPE-Overall; RPE-Chest; RPE-Legs) and affect (Feeling Scale) were recorded at 200 m intervals during the TT. Lastly, RPE and affect for the entire exercise session (session RPE and session affect) were obtained 5 min following the one-mile TT. Expired respiratory gases were continuously collected during the entire time trial and capillary blood was collected Pre- and Post-TT.

### Repeated sprint performance

Participants arrived at the laboratory in the morning at least ≥ 3 h post-prandial. Upon arrival to the laboratory subjects completed a 10 min self-paced warm-up on the treadmill. The RSP prolonged high intensity interval protocol was chosen because it requires a high level of aerobic oxidative, as well as anaerobic glycolytic contributions (50). The RSP was performed on a treadmill and consisted of 6 × 800 m (0.5 miles) sprints, with a three-minute passive recovery interval. Subjects were instructed to finish each 800 m sprint as fast as possible. The time it takes to complete each sprint was recorded. No feedback on sprint times was given to the participants during trials. Verbal encouragement was provided during maximum effort sprints in a standardized fashion throughout each visit. Heart rate, blood samples, RPE, affect, and metabolic gases were collected throughout.

### Respiratory gas exchange

Respiratory gas exchange was recorded using an automated metabolic analyzer system (TrueOne 2400, ParvoMedics, Sandy, UT, United States). Prior to each experimental session, the device was calibrated using procedures according to manufacturer instructions. The breath-by-breath measurements were performed for oxygen uptake (VO_2_), carbon dioxide production (VCO_2_), and respiratory exchange ratio (RER) and was measured continuously throughout trials (one-mile TT and RSP). The average values for VO_2_ (L/min) and VCO_2_ (L/min) were calculated over the last minute of each 2-min exercise stage in the maximal exercise test, and each minute of the one-mile TT. Whole-body rates (g/min) of CHO and fat oxidation were calculated using intensity dependent equations that assume negligible protein contribution to energy expenditure (51).

### Blood metabolites

Blood samples were measured via fingertip blood samples collected using a lancet following cleaning of the fingertip with an alcohol swab and then dried. The first droplet was wiped away with a cotton swab to remove any alcohol and the subsequent droplets were used for analysis. Samples were immediately processed for measurement of blood lactate (Lactate Plus, Nova Biomedical), ketones (*R*-β-hydroxybutyrate; Precision Xtra, Abbott Diabetes Care Inc., Almeda, CA) and glucose (Precision Xtra, Abbott Diabetes Care Inc., Almeda, CA) concentrations.

### Biochemical assays

Blood samples were collected using a validated dried blood spot (DBS) card technique (52). DBS cards were allowed to dry for 30 min, and then stored at −30°C prior to shipment to ZRT CLIA-approved Laboratory (Beaverton, OR) for immunoassay analyses as previously described (52, 53). DBS were assayed for HbA_1c_, total cholesterol, low-density lipoprotein cholesterol (LDL-C), very low-density lipoprotein cholesterol (VLDL-C), high-density lipoprotein cholesterol (HDL-C), triglycerides, insulin, and high-sensitivity C-reactive protein (hsCRP). Dried blood spot testing has shown a strong correlation with conventional serum tests, making it a reliable and convenient tool for screening cardiometabolic risk factors (52).

### Continuous glucose monitoring

Participants’ interstitial glucose concentrations were measured throughout each 31-day dietary intervention via CGM (Freestyle Libre 2, Abbott Diabetes Care Inc; Almeda, CA) utilizing Levels, Inc., mobile application software to capture glucose response and trends in real-time (Levels Health, Levels, Inc., New York, New York). CGM tracks long-term to HbA_1c_ (54–56), short term CGM readings (10-14d) are good estimates of 3-month CGM averages (57), and (iii) CGM can also capture both fasting and post-prandial differences in glucose (validated diagnostic tools). Participants were instructed to apply a 14-day sensor to the back of the arm following the manufacturer’s written instructions and demonstration video. Participants were instructed to place it in the same location throughout. CGM subcutaneous sensor measured interstitial glucose values every 15 min which was transmitted using near-field communications. Participants obtained their glucose values after scanning for up to 8 h of data. All 24-hour CGM readings were included into daily and 31-day statistics. All CGM sensors were obtained in batch to control for any potential manufacturing discrepancies across different sensor batches.

### Statistical analysis

Statistical analyses were performed using SPSS version 26.0 (SPSS Inc., Chicago, IL), and MATLAB 2022a (MathWorks Inc., Natick, MA). Statistical significance was set *a priori* at *p* < 0.05. Descriptive statistics were calculated for all variables. Normality and absence of large outliers were verified by using the Shapiro-Wilks test, observing the normality plots, and residual plots. Repeated measure analyses of variance (ANOVA) were utilized to assess physiologic, metabolic, respiratory, perceptual, and performance time, treatment, and interaction effects. Bonferroni *post hoc* was utilized to control for multiple comparisons when main effects were observed. A paired samples *t*-test was used to analyze differences in macronutrient composition and 31-day glycemic variable averages between the two dietary interventions. A one-way repeated measures analysis of variance was used to analyze differences over time for training load. The assumption of sphericity was confirmed using Mauchly’s test. Greenhouse-Geisser epsilon corrections were used when the sphericity assumption was violated. Partial-eta squared (η2p) was used to report the effect sizes for the above metrics, where appropriate. Simple linear regression analyses were run to determine the relationship between glycemic, substrate oxidation, and biochemical parameters. Slope intercept (y = mx + b), r^2^, and p values represent best-fit equation, the goodness of fit, and slope significantly non-zero values, respectively. To avoid (58) assumptions of normality in continuous data, a kruskal Wallis (KW) test with Dunn’s correction for multiple comparisons was utilized to determine differences across the entire 24 h circadian window for the all data in the 31d CGM response window. Time points every 4th h across the entire 24 h period were included in the statistical comparison, corresponding to ∼1 point maximum per ultradian cycle (59, 60). All data are reported as Median ± SD, with exception of circadian glucose patterns presented as Median, 25th, and 75th percentile. Sample size (*n* = 10) was determined based on prior studies where one-mile TT performance differences were observed using dietary interventions in elite runners which was adjusted for the expected increase in one-mile TT performance time variance across cohort in non-elite athletes (61).

## Results

### Dietary and exercise adherence

Daily dietary nutrient intakes are summarized in Table 1. All participants (*n* = 10) completed all the required study duties. The energy intake between LCHF and HCLF treatments remained isocaloric. Significant differences were detected for every diet composition variable, notably for carbohydrate and fat intake. LCHF consumed less absolute and relative carbohydrates compared to HCLF, largely driven by the 10-fold reduction in simple sugars and approximately one-half the dietary fiber. Conversely, LCHF consumed significantly more dietary fat and cholesterol, both as absolute and relative amounts, compared to HCLF. While attempts were made to control protein intake across groups, athletes consumed an extra 31 grams of protein during the LCHF treatment (*p* < 0.01), likely due to the close association between dietary fat and protein.

**FIGURE 3:**
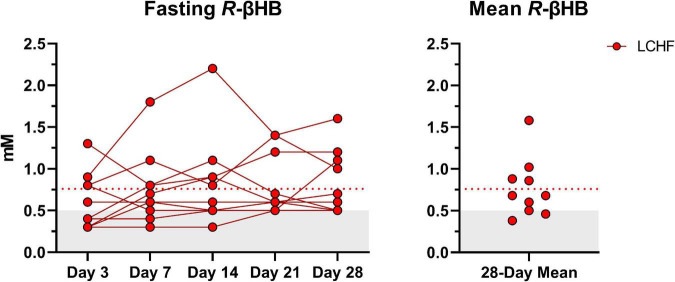
Daily capillary ketones. *y*-axis values ≥ 0.5 mM define a range characterized as nutritional ketosis. The dotted line denotes the low carbohydrate high fat (LCHF) *R*-beta-hydroxybutyrate (*R*-βHB) mean. Eight out of ten participants consistently remained in nutritional ketosis throughout the LCHF intervention, whereas two participants were marginally below threshold. One-Way ANOVA revealed that there were no significant time effects from day 3 and thereafter, meaning that ketosis was rapidly induced and maintained over four-weeks. *n* = 10.

Average capillary *R*-βHB (mean ± SD: 0.76 ± 0.04 mM; range: 0.3 – 2.2 mM) during LCHF increased significantly from baseline and remained within the range of nutritional ketosis (≥ 0.5 mM *R*-βHB) throughout the intervention (Figure 3).

There were no significant differences for weekly training load, either within- or between dietary phases (Table 2).

**TABLE 2:** Training load.

	Diet treatment	Week 1	Week 2	Week 3	Week 4	Grand mean	Within-diet effect (*P*-value)	Between-diet effect (*P*-value)
Training Load (RPE x min)	LCHF	2928 ± 2057	2543 ± 2172	2343 ± 1822	2143 ± 1917	2489 ± 2425	0.14	0.94
	HCLF	2077 ± 1161	2630 ± 1507	2489 ± 1669	2503 ± 1488	2425 ± 1398	0.18	

Weekly training load were captured on low carbohydrate high fat (LCHF) and high carbohydrate low fat (HCLF) diets. 2 (Treatment) x 4 (Time) ANOVA revealed no significant differences in training load. *n* = 10. Values are Mean ± SD. RPE, rate of perceived exertion. LCHF = low-carbohydrate/high-fat diet; HCLF = high-carbohydrate/low-fat diet.

### Body composition data

Participants started each diet at similar weight and body composition. There were no significant treatment or interaction effects for weight or body composition during either LCHF or HCLF (Table 3). Overall changes in weight and body composition on each diet were similar. Significant time effects were detected for weight and BMI in both LCHF and HCLF treatments. Approximately ∼80% of weight loss composition was derived from fat mass (Δ: 1.8 ± 0.6 kg; *p* = 0.05) in 4:1 ratio fat:fat-free mass, while the remaining portion of weight loss was derived non-significantly from fat-free mass (Δ: 0.6 ± 0.2 kg).

**TABLE 3:** Body composition.

Variable	LCHF			HCLF			*P-value*; η 2p		
	Pre Day –4	Post Day 28	Change Mean (95% CI)	Pre Day –4	Post Day 28	Change Mean (95% CI)	Treatment	Time	Interaction
**Mean data**									
Weight (kg)	85.2 ± 12.5	81.9 ± 10.9**^#^**	−3.2 (5.2, 1.3)	84.8 ± 10.2	83.4 ± 9.4**^#^**	−1.4 (3.4, −0.6)	0.676; 0.020	**0.012**; 0.519	0.071; 0.318
BMI (kg/m^2^)	25.6 ± 2.23	24.7 ± 1.99**^#^**	−0.89 (1.5, 0.2)	25.7 ± 2.38	25.3 ± 1.84**^#^**	−0.44 (1.0, −0.1)	0.379; 0.087	**0.017**; 0.487	0.165; 0.202
Body Fat (%)	14.5 ± 4.80	11.9 ± 3.90	−2.5 (5.2, −0.2)	13.3 ± 5.14	13.0 ± 3.84	−0.26 (1.5, −1.0)	0.938; 0.001	0.080; 0.302	0.104; 0.267
Fat Mass (kg)	12.7 ± 5.26	9.99 ± 3.97**^#^**	−2.7 (5.3, 0.0)	11.5 ± 5.00	10.7 ± 4.02**^#^**	−0.78 (−2.2, 0.7)	0.849; 0.040	**0.050**; 0.363	0.127; 0.239
Fat Free Mass (kg)	72.5 ± 8.69	71.9 ± 8.74	−0.61 (2.3, −1.1)	73.3 ± 8.21	72.7 ± 8.15	−0.63 (1.6, −0.4)	0.111; 0.258	0.256; 0.140	0.976; 0.000

Body composition was assessed using bioelectrical impedance pre and post low-carbohydrate/high-fat (LCHF) and high-carbohydrate/low-fat diet (HCLF). 2 (Treatment) x 2 (Time) Repeated Measures ANOVA demonstrated no differences across treatments. A main effect of time was observed within LCHF and HCLF treatments for weight, body mass index (BMI) and fat mass (FM), and a trend in body fat percentage. Fat free mass (FFM) did not change over time. No significant interaction effects were observed, although trends were observed across LCHF and HCLF for weight (kg; *p = 0.071*). *n* = 10. Mean ± SD.

^#^ = significant difference for Pre vs. Post within-diet (*p* ≤ 0.05). Bold values represent the *p* < 0.05.

### Physiological, metabolic, respiratory, perceptual, and performance data collected during the one-mile time trial

All 10 participants completed the treadmill one-mile time trial (1,609 m) at a self-selected pace before initiating either diet. No significant baseline (Pre) differences were observed across physiological, metabolic, respiratory, perceptual, or performance parameters. Significant differences were detected within-diet and between-treatments at the post-timepoint (Table 4).

**TABLE 4:** One-mile time trial performance, gas exchange, and perception.

Variable	LCHF			HCLF			*P-value*; η 2p		
	Pre Day –4	Post Day 28	Change Mean (95% CI)	Pre Day –4	Post Day 28	Change Mean (95% CI)	Treatment	Time	Interaction
Time (sec)	381.4 ± 30.6	367.1 ± 37.5**^#^**	−14.3 (26.4, 2.2)	374.1 ± 38.6	374.0 ± 31.5**^#^**	–0.10 (11.1, –10.9)	0.958; 0.000	**0.009**; 0.553	0.159; 0.208
Mean Carbohydrate Oxidation (g/min)	5.22 ± 1.07	4.16 ± 1.48^†^	−1.06 (1.8, 0.3)	5.50 ± 2.14	5.91 ± 1.07	0.4 (0.7, –1.5)	0.099; 0.273	0.335; 0.103	**0.028**; 0.433
Mean Fat Oxidation (g/min)	0.23 ± 0.29	0.67 ± 0.45^#^*^†^	0.44 (−0.3, −0.6)	0.21 ± 0.29	0.05 ± 0.11**^#^**	–0.15 (0.32, –0.01)	**0.023**; 0.454	**0.029**; 0.427	**0.001**; 0.756
Heart Rate (b.min-1)	168.8 ± 8.7	173.4 ± 8.8^#^*^†^	4.46 (−0.8, −8.2)	167.7 ± 10.8	167.1 ± 11.1	–0.59 (4.2, –2.9)	**0.003**; 0.644	0.145; 0.220	**0.040**; 0.389
Mean VO_2_ (ml/kg/min)	50.2 ± 5.63	52.9 ± 6.84	2.72 (0.5, −5.9)	48.4 ± 8.98	50.0 ± 6.19	1.67 (2.7, –6.0)	0.070; 0.319	0.116; 0.252	0.651; 0.024
Mean VO_2_ (L/min)	4.23 ± 0.44	4.34 ± 0.66*	0.11 (0.1, −0.3)	4.03 ± 0.62	4.17 ± 0.62*	0.14 (0.2, –0.5)	**0.013**; 0.516	0.257; 0.140	0.869; 0.003
Mean VCO_2_ (L/min)	4.13 ± 0.46	3.97 ± 0.64	−0.15 (0.4, −0.1)	4.05 ± 0.82	4.24 ± 0.61	0.19 (0.3, –0.7)	0.580; 0.035	0.904; 0.002	0.119; 0.249
Mean V_E_ (L/min)	122.5 ± 13.4	123.6 ± 18.5	1.13 (6.1, −8.4)	116.8 ± 16.9	123.4 ± 15.0	6.67 (7.3, –20.6)	0.296; 0.120	0.332; 0.105	0.396; 0.081
Mean RR (bpm)	41.7 ± 6.47	43.9 ± 5.83	2.19 (−0.1, −4.3)	42.0 ± 3.98	42.9 ± 6.52	0.93 (3.0, –4.9)	0.712; 0.016	0.154; 0.212	0.534; 0.045
Mean RER	0.97 ± 0.05	0.91 ± 0.07*^†^	−0.06 (0.10, 0.01)	1.00 ± 0.09	1.01 ± 0.05*	0.01 (0.03, –0.06)	**0.045**; 0.376	0.110; 0.259	**0.021**; 0.466
Session RPE	7.40 ± 1.64	7.90 ± 1.59	0.5 (0.3, −1.3)	7.60 ± 1.42	7.70 ± 1.49	0.1 (0.8, –1.0)	1.000; 0.000	0.081; 0.300	0.574; 0.036
RPE-O	6.70 ± 1.14	6.72 ± 1.02	0.02 (0.4, −0.5)	6.64 ± 0.89	6.69 ± 1.09	0.05 (0.5, −0.6)	0.871; 0.003	0.868; 0.003	0.897; 0.002
Session affect	−0.10 ± 2.96	−0.30 ± 2.98	−0.2 (0.5, −0.1)	0.00 ± 3.01	−0.50 ± 2.87	−0.50 (2.1, −1.1)	0.935; 0.001	0.322; 0.109	0.703; 0.017
Affect	0.04 ± 2.16	0.51 ± 1.93**^#^**	0.47 (0.15, −1.1)	0.41 ± 1.73	0.79 ± 1.76**^#^**	0.38 (0.2, −1.0)	0.379; 0.087	**0.017**; 0.487	0.852; 0.004

One-mile time trial performance, gas exchange, and perception metrics were gathered pre and post-low carbohydrate high fat (LCHF) and high carbohydrate low fat treatments. n = 10. Mean ± SD. RPE-O = RPE for overall body; RPE = rating of perceived exertion (OMNI rating of exertion); RER = Respiratory exchange ratio; VO_2_ = oxygen consumption; VCO_2_ = carbon dioxide production; VE = ventilation; RR = Respiratory Rate; LCHF = low carbohydrate high fat diet; HCLF = high carbohydrate low fat diet.

^#^ = significant difference from Pre vs. Post (*p* ≤ 0.05).

* = significant difference between treatment (*p* ≤ 0.05); **^†^** = significant difference between LCHF and HCLF at Post (*p* ≤ 0.05). Bold values represent the *p* < 0.05.

Within-diet, LCHF mean respiratory exchange rate (Δ: −0.08 ± 0.02; −6%), and mean carbohydrate oxidation (Δ:−1.06 ± 0.36 g/min; −20%) decreased significantly from Pre to Post, whereas heart rate (Δ: 4 ± 1 bpm; 3%) and mean fat oxidation (Δ: 0.44 ± 0.16 g/min; 190%) increased significantly from pre to post (all *p* < 0.05). The significant decrease in respiratory exchange rate from Pre to Post-LCHF revealed a decrease in carbohydrate reliance (91 to 73%) and an increase in fat utilization (9 to 27%). Additionally, these effects were corroborated by the absolute (g/min) carbohydrate and fat oxidation rates, which revealed that for every 1 g/min decrease in carbohydrate oxidation there was an expected 0.42 g/min of increase in fat oxidation during LCHF. There were no significant changes in substrate oxidation and respiratory exchange rate detected pre- to post-HCLF.

Significant interactions revealed that Post-LCHF athletes had a higher heart rate (Δ: 6 ± 2 bpm), mean fat oxidation rate (Δ: 0.62 ± 0.21 g/min), mean respiratory rate (Δ: 1.0 ± 0.3 bpm), and a lower carbohydrate oxidation rate (Δ: 1.75 ± 0.60 g/min) compared to Post-HCLF (all *p* < 0.05).

### Physiological, metabolic, espiratory, perceptual, and performance data collected during the repeated sprint protocol

**FIGURE 4:**
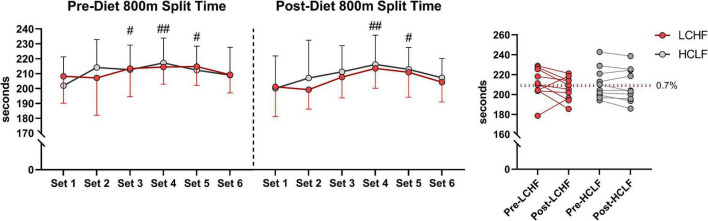
Repeated sprint running performance. Diet did not significantly alter running times between or within treatments. There was a main effect of time for running performance reflected in higher running split time at set 3, 4, and 5 during pre-diet phase. Post-diet phase showed significant increase in split time during set 4 and 5 only. *n* = 10. Mean ± SD. LCHF = low-carbohydrate/high-fat diet; HCLF = high-carbohydrate/low-fat diet. ^#^, ^##^ are *p* < 0.05 and 0.01 significantly different from Set 1, respectively.

All 10 participants completed the treadmill series of 6-sets of 800m sprints at a self-selected pace. Before each dietary phase there were no significant differences in physiological, metabolic, respiratory, perceptual, or performance parameters. Additionally, neither diet influenced repeated sprints running performance post-diet intervention (Table 5 and Figure 4).

**TABLE 5:** Repeated sprint protocol performance, gas exchange, and perception.

Variable	LCHF			HCLF			*P-value*; η 2p		
	**Pre** **Day –1**	**Post** **Day 31**	**Change** **Mean (95% CI)**	**Pre** **Day –1**	**Post** **Day 31**	**Change** **Mean (95% CI)**	**Treatment**	**Time**	**Interaction**
Total time (sec)	1267.0 ± 90.3	1236.1 ± 69.2	–30.9 (84.9, –23.1)	1267.1 ± 93.3	1254.0 ± 101.2	–13.1 (32.2, –6.0)	0.695; 0.018	0.064; 0.331	0.556; 0.040
Mean carbohydrate oxidation (g/min)	3.13 ± 1.08	1.44 ± 0.84^#^*^†^	–1.69 (2.7, 0.7)	3.66 ± 1.19	3.66 ± 0.52^#^*	–0.00 (0.8, –0.8)	**0.004**; 0.611	**0.011**; 0.532	**0.023**; 0.456
Mean fat oxidation (g/min)	0.75 ± 0.36	1.44 ± 0.41^#^*^†^	0.69 (–0.3, –1.1)	0.61 ± 0.31	0.53 ± 0.22^#^*	–0.08 (0.4, –0.2)	**0.002**; 0.683	**0.012**; 0.526	**0.007**; 0.577
Heart rate (b.min-1)	160.5 ± 9.2	163.2 ± 13.5	2.64 (2.7, –8.0)	162.0 ± 7.6	159.6 ± 8.4	–2.44 (5.0, –0.2)	0.700; 0.017	0.931; 0.001	0.125; 0.242
Mean VO_2_ (ml/kg/min)	45.8 ± 5.75	48.9 ± 3.89	3.09 (–0.3, –5.9)	46.4 ± 8.02	46.1 ± 5.15	–0.31 (3.8, –3.2)	0.457; 0.063	0.259; 0.139	0.070; 0.320
Mean VO_2_ (L/min)	3.87 ± 0.41	3.99 ± 0.41	0.12 (0.1, –0.3)	3.86 ± 0.49	3.80 ± 0.50	–0.06 (0.3, –0.2)	0.366; 0.091	0.662; 0.022	0.191; 0.182
Mean VCO_2_ (L/min)	3.34 ± 0.39	3.20 ± 0.35	–0.14 (0.5, –0.2)	3.53 ± 0.57	3.50 ± 0.42	–0.03 (0.3, –0.2)	0.083; 0.297	0.388; 0.084	0.504; 0.051
Mean V_E_ (L/min)	107.0 ± 10.4	106.8 ± 11.3	–0.18 (5.0, –4.7)	106.9 ± 13.0	106.7 ± 10.2	–0.24 (8.7, –8.2)	0.960; 0.000	0.918; 0.001	0.990; 0.000
Mean RR (bpm)	40.8 ± 7.51	39.7 ± 6.88	–1.08 (2.6, –0.5)	40.3 ± 7.82	40.7 ± 7.38	0.41 (2.1, –2.9)	0.796; 0.008	0.627; 0.027	0.272; 0.132
Mean RER	0.86 ± 0.05	0.80 ± 0.04^#^*^†^	–0.06 (0.15, 0.05)	0.91 ± 0.05	0.92 ± 0.04^#^*	0.01 (0.0, –0.1)	**0.001**; 0.716	**0.005**; 0.596	**0.010**; 0.536
Session RPE	7.20 ± 1.03	7.10 ± 1.37	–0.10 (0.8, –0.6)	7.10 ± 0.99	7.60 ± 1.26	0.50 (0.0, –1.0)	0.534; 0.044	0.399; 0.080	0.081; 0.300
RPE-O	7.16 ± 0.93	6.87 ± 1.08	–0.29 (0.7, –0.1)	6.91 ± 0.84	7.16 ± 0.75	0.25 (0.3, –0.8)	0.927; 0.001	0.894; 0.002	0.095; 0.279
Session affect	0.50 ± 2.55	0.00 ± 2.75**^#^**	–0.50 (1.1, –0.1)	0.30 ± 3.05	–0.10 ± 3.07**^#^**	–0.40 (1.1, –0.3)	0.678; 0.020	**0.041**; 0.386	0.823; 0.006
Affect	0.16 ± 2.52	0.18 ± 2.59	0.02 (0.8, –0.9)	0.34 ± 2.45	–0.01 ± 2.57	–0.35 (0.9, –0.2)	0.984; 0.000	0.347; 0.099	0.507; 0.050

Repeated sprint protocol performance, gas exchange, and perception after low carbohydrate high fat (LCHF) and high carbohydrate low fat (HCLF) treatments. LCHF lowered mean carbohydrate and respiratory exchange ratio, and lowered mean fat oxidation but remained unchanged pre to post-HCLF. Both LCHF and HCLF lowered session affect. *n* = 10. Mean ± SD. Total time = time to complete all sprints; RPE-O = RPE for overall body; RPE = rating of perceived exertion (OMNI rating of exertion); RER = Respiratory exchange ratio; VO_2_ = oxygen consumption; VCO_2_ = carbon dioxide production; V_E_ = ventilation; RR = Respiratory Rate; LCHF = low carbohydrate high fat diet; HCLF = high carbohydrate low fat diet.

^#^ = significant difference from Pre vs. Post (*p* ≤ 0.05).

* = significant difference between treatment (*p* ≤ 0.05); **^†^** = significant difference between LCHF and HCLF at Post (*p* ≤ 0.05). Bold values represent the *p* < 0.05.

**FIGURE 5:**
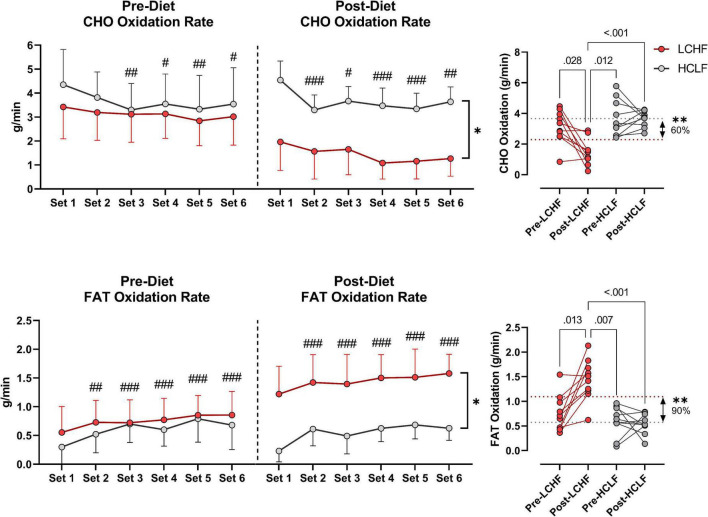
Substrate oxidation rates. Carbohydrate (CHO) and lipid (FAT) oxidation rates (g/min) were calculated using intensity-dependent formulas. Pre-diet CHO and FAT oxidation were comparable between the high-carbohydrate/low-fat (HCLF) and low-carbohydrate/high-fat (LCHF) treatment from set 1 and thereafter. There was a main effect of time for CHO and FAT oxidation reflected in lower CHO (*p* < 0.05) and higher FAT (*p* < 0.001) oxidation by set 6, respectively. Post-diet CHO and FAT oxidation were significantly altered by diet. Within-LCHF treatment there was a significant decrease in CHO oxidation (–54%; p < 0.05) and a significant increase in FAT oxidation (93%; p < 0.05) from Pre to Post-diet. No Pre-Post substrate oxidation changes were detected for HCLF. *n* = 10. Mean ± SD. ^#^, ^##^, and ^###^ are *p* < 0.05, 0.01, and 0.001 significantly different from Set 1, respectively. *, ^**^ are *p* < 0.05, 0.01 significantly different between diets, respectively.

During RSP, LCHF induced very high rates of fat oxidation which peaked at 1.58 ± 0.33g/min during 86.40 ± 6.24%VO_2max_ (range: 0.99 to 2.01 g/min) compared to 0.69 ± 0.24g/min on HCLF during 79.67 ± 3.15%VO_2max_ (range: 0.32 to 1.13 g/min). Interestingly, 30% of the subjects on LCHF had peak fat oxidation rates > 1.85 g/min (1.95 ± 0.08 g/min; range: 1.86 to 2.01 g/min). To our knowledge, these are the highest rates of fat oxidation ever recorded. Within-diet, LCHF mean respiratory exchange rate (Δ:−0.06 ± 0.02; −7%), and mean carbohydrate oxidation (Δ:−1.69 ± 0.57 g/min; −54%) decreased significantly from pre to post, whereas mean fat oxidation (Δ: 0.69 ± 0.23 g/min; 92%) increased significantly from pre to post (all *p* < 0.05). The significant decrease in respiratory exchange rate indicated decreased carbohydrate reliance (53 to 35%) toward fat utilization (47 to 65%). Additionally, the absolute carbohydrate and fat oxidation rates (g/min) demonstrated that for every 1 g/min decrease in carbohydrate oxidation there was 0.41 g/min increase in fat oxidation. No significant Pre-Post changes were detected within-HCLF (Figure 5 and Supplementary Table 1).

There was a trend for greater mean relative VO_2_ consumption (*p* = 0.07) and lower rate-of-perceived exertion, both as overall body RPE-O (*p* = 0.095) and session RPE (*p* = 0.08), when comparing post-LCHF to post-HCLF. This increased relative oxygen consumption after LCHF is largely explained by weight-loss, and partly explained by the non-significant increase in heart rate (i.e., greater cardiac output) and fat oxidation, altogether increasing tissue demand for oxygen and thereby augmenting relative VO_2_.

### Blood metabolite data

#### One-mile time trial (TT; 1,609 m)

Prior to dietary intervention, capillary blood *R*-βHB was below the limit of nutritional ketosis pre-diet and pre-TT (mean ± SD: 0.2 ± 0.1 mmol/L) and remained unaltered pre-diet post one-mile TT across groups. Post-LCHF treatment significantly increased *R*-βHB from baseline (0.21 ± 0.11 vs. 0.67 ± 0.30 mmol/L *R*-βHB; *p* < 0.001). *R*-βHB concentrations demonstrated a slight decrease after exercise, however, the effect was non-significant (Δ = −0.14 ± 0.10 mmol/L; *p* = 0.23).

Capillary blood glucose concentrations were similar Pre- and Post-diet between LCHF and HCLF treatments (LCHF average: 87.4 ± 9.6 mg/dL vs. HCLF average: 90.7 ± 10.5 mg/dL; *p* = 0.27) Pre-one-mile TT. There was a main effect of time observed Post one-mile TT that raised blood glucose from baseline (89.1 ± 10.0 to 126.8 ± 20.2 mg/dL; + 42%; *p* < 0.001), independent of diet.

**FIGURE 6:**
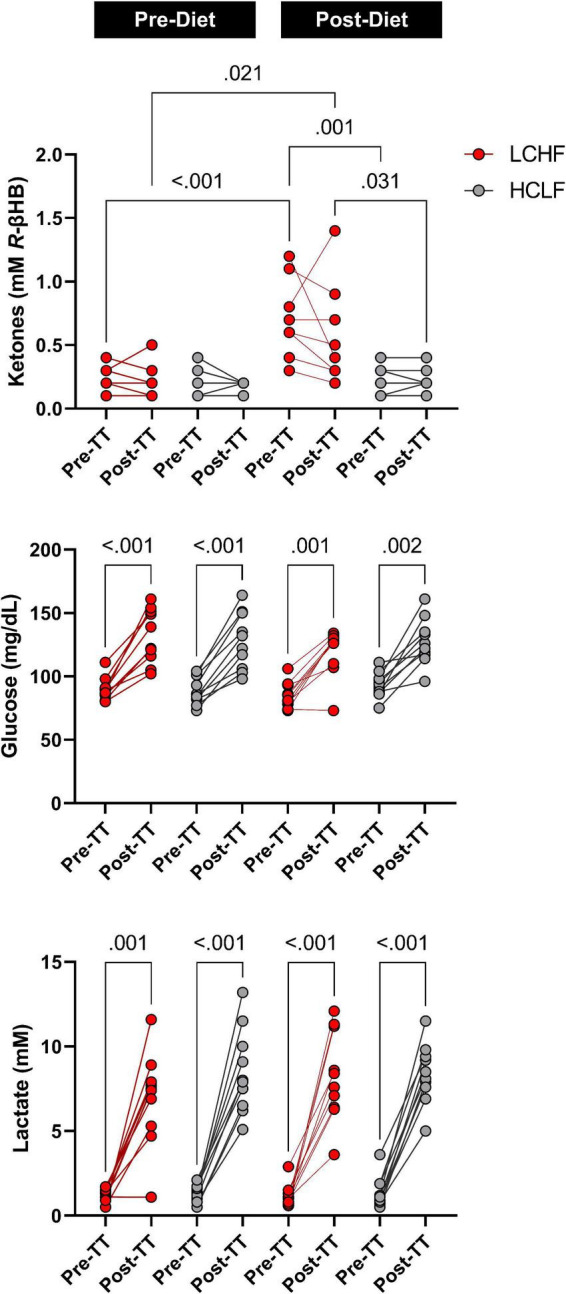
One-mile time-trial metabolite impact. Capillary ketones, glucose and lactate were measured immediately pre- and post-one mile time trial (TT) to evaluate the within- and between-diet effects in the context of exercise. There were no significant Pre-diet and Pre-TT differences. A significant post-diet effect was detected in capillary ketones Pre-TT. Glucose and lactate were significantly elevated over time, independent of diet and dependent on TT. *n* = 10. Mean ± SD. LCHF = low-carbohydrate/high-fat diet; HCLF = high-carbohydrate/low-fat diet.

Capillary blood lactate before the one-mile TT was at the same concentration Pre- and Post-diet in both LCHF and HCLF treatments (LCHF average: 1.1 ± 0.5 vs. HCLF average: 1.3 ± 0.7 mmol/L; *p* = 0.48). There was a main effect of time induced by exercise that increased blood lactate significantly from baseline (1.2 ± 0.6 to 8.0 ± 2.4 mg/dL; + 557%; *p* < 0.001), independent of diet (Figure 6 and Supplementary Table 2).

#### Repeated sprint protocol (RSP; 6 × 800 m)

Capillary blood *R*-βHB was below the limit of nutritional ketosis Pre-diet and Pre-RSP (0.14 ± 0.05 mmol/L). LCHF significantly increased *R*-βHB into nutritional ketosis compared to pre-diet concentrations (0.19 ± 0.12 vs. 0.72 ± 0.73 mmol/L; 279%; *p* < 0.001) whereas HCLF did not meaningfully influence *R*-βHB. Over the course of the RSP, *R*-βHB decreased by approximately 0.05 mmol/L between sets and was significantly lower from Pre- to Post-RSP (Δ = −0.26 ± 0.09 mmol/L; *p* = 0.001), denoting increased ketone oxidation rates after every sprint.

Capillary blood glucose concentrations Pre-diet and Pre-RSP (99.9 ± 6.2 mg/dL) increased significantly by set 5 (106.2 ± 25.7 mg/dL; *p* = 0.012) and Post (107.2 ± 26.4 mg/dL; *p* = 0.006). There was a treatment-dependent effect that revealed lower blood glucose levels during LCHF diet compared to HCLF diet (Δ = −8.5 ± 3.8 mg/dL; 8%; *p* = 0.03).

**FIGURE 7:**
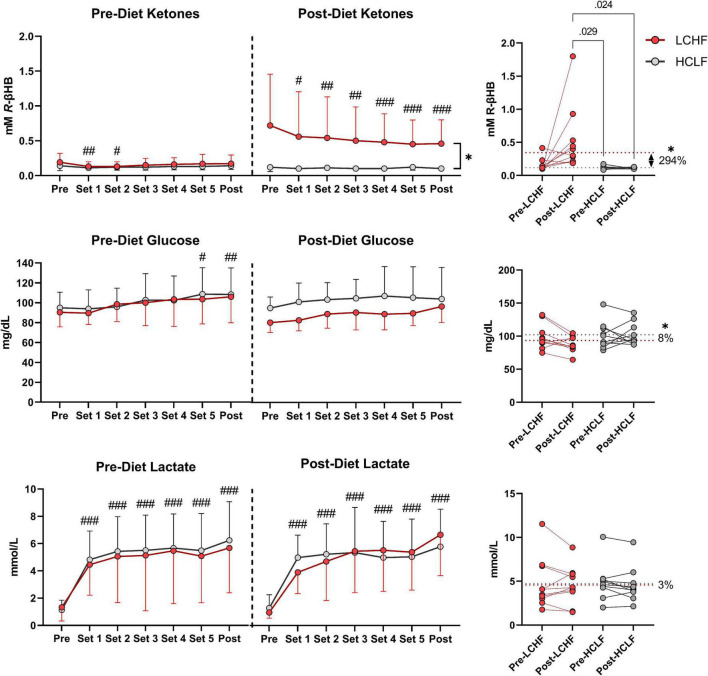
Repeated sprint performance metabolite impact. Capillary R-βHB, glucose, and lactate were measured in capillary blood immediately after each repeated sprint performance (RSP) set. There were no significant between-treatment differences pre-diet. The significant time effects were detected in lower R-βHB values at set 1 and 2, higher glucose values at set 5 and post, and significantly higher lactate values from set 1 and thereafter. Post-diet ketones were significantly influenced by low carbohydrate high fat (LCHF) treatment, with 3-fold higher R-βeta-Hydroxybutyrate (R-βHB) concentrations throughout the sets compared to HCLF. *R*-βHB decreased between each set by a total of 46% from pre-post (*p* < 0.001). Glucose was overall 8% lower during LCHF compared to HCLF (*p* = 0.03). Lactate was not affected significantly by diet. *n* = 10. Mean ± SD. ^#^, ^##^, and ^###^ are *p* < 0.05, 0.01, and 0.001 significantly different from the Pre timepoint. * is *p* < 0.05 between diet overall effect.

Capillary lactate concentrations Pre-diet and Pre-RSP (1.2 ± 0.9 mmol/L) increased significantly after set 1 (4.6 ± 2.2 mmol/L; *p* = 0.001) and thereafter. Peak lactate concentrations (6.0 ± 2.8 mmol/L; *p* = 0.001) were recorded at set 6 (i.e., post-set). Diet did not significantly influence the rate of lactate appearance in the blood nor peak lactate (Figure 7 and Supplementary Table 3).

### Cardiometabolic indices

**FIGURE 8:**
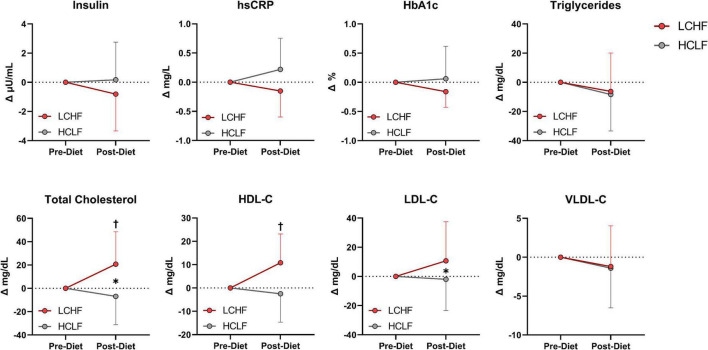
Cardiometabolic scores. Statistics were conducted on absolute values and are presented as mean change from Pre-diet. There were no cardiometabolic differences Pre-diet or significant time effects. Between-diet effects revealed greater total cholesterol and LDL-C concentrations during the LCHF versus HCLF treatment. The significant interaction revealed greater total cholesterol and HDL-C concentrations Post-LCHF treatment. *n* = 10. Mean ± SD. * = *p* < 0.05 between-diet main effect. † = significant interaction Post-diet (*p* < 0.05).

There were no significant changes over time in any of the variables of interest (Figure 8 and Supplementary Table 4). Between-condition effects reveal higher total cholesterol (Δ: 20.7 ± 3.3 mg/dL; *p* = 0.001) and LDL-C (Δ: 10.7 ± 4.6 mg/dL; *p* = 0.03) after LCHF. Interaction effects revealed total cholesterol (Δ: 29.5 ± 6.3 mg/dL; *p* = 0.007) and HDL-C (Δ: 11.4 ± 3.3 mg/dL; *p* = 0.045) were significantly greater Post-diet on LCHF treatment.

### Glycemic control

**FIGURE 9:**
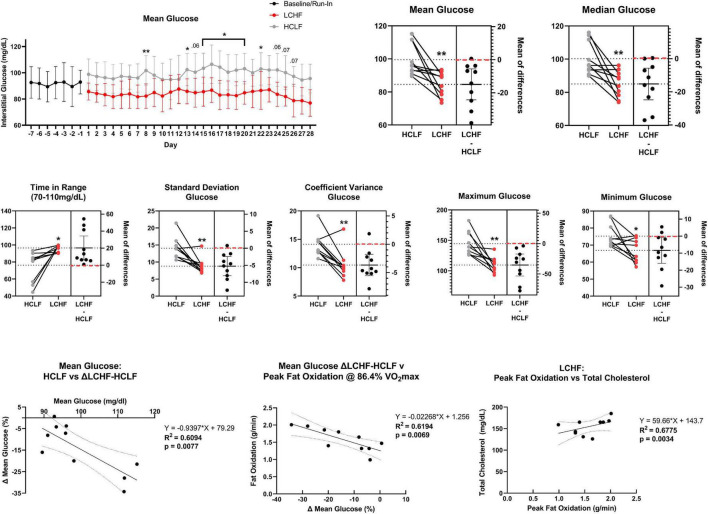
Continuous glucose monitoring. Interstitial glucose values were continuously gathered every 15 min over the duration of the sensor life prior to treatment (baseline/run-in), and during both low carbohydrate high fat (LCHF) and high carbohydrate low fat (HCLF) diets. Average glucose was significantly lower on LCHF on day 8, 13, 15-20, and 22. All glycemic parameters over the 31-days dietary intervention were significantly improved during LCHF. Across all variables, only 0-2/10 subjects favored HCLF. The 31-day mean glucose predicted the percent change in mean glucose between LCHF and HCLF diets. 30% of subjects had mean and median glucose > 100 mg/dL throughout HCLF treatment. These subjects were the largest responders to LCHF with no subject > 100 mg/dL during LCHF treatment. These same subjects also reported the highest peak fat oxidation rate as the percent change in mean glucose between LCHF and HCLF diets predicted the peak oxidation rates across the entire cohort. Peak fat oxidation rates on LCHF were also associated with higher cholesterol demonstrating a potential interaction between oxidation rates and global lipid metabolism. *n* = 10. Mean ± SD. *, ** and *p* < 0.05, *p* < 0.01 are significant difference between LCHF and HCLF at same time point.

**FIGURE 10:**
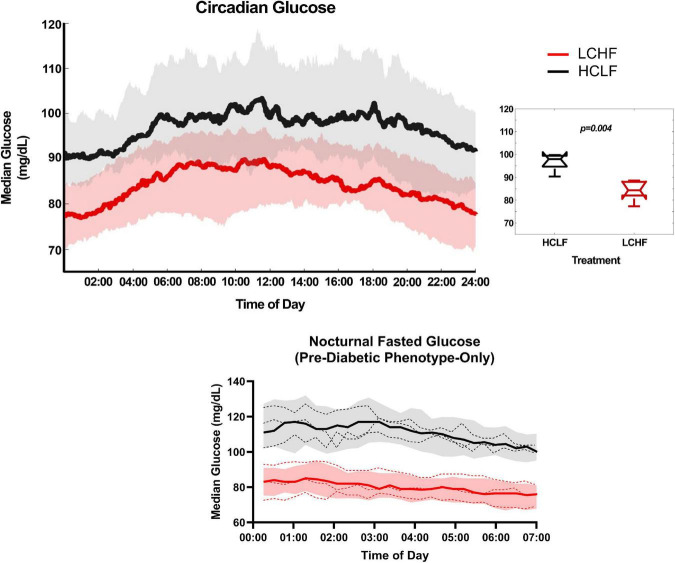
Circadian glucose patterns. Interstitial glucose values were continuously gathered every 15 minutes over the duration of the sensor life prior to treatment (baseline/run-in), and during both low carbohydrate high fat (LCHF) and high carbohydrate low fat (HCLF) diets. 31-day glucose values were plotted over 24-h time of day (circadian glucose patterns). Median glucose was significantly lower across the entire day during LCHF (*p* = 0.004). 31-day nocturnal fasting glucose (00:00 to 07:00) for the 30% of subjects with pre-diabetic phenotype (mean and median glucose > 100 mg/dL), all had fasting glucose values > 100 mg/dL. These subjects with pre-diabetic phenotype all reduced their fasting glucose < 100 mg/dL on LCHF. *n* = 10. Median ± 25 to 75% Percentiles. Red, LCHF. Black, HCLF. Dashed line, individual pre-diabetic subject circadian glucose patterns.

All glycemic parameters significantly improved on LCHF (Figures 9, 10). Average glucose was significantly lower during LCHF treatment starting day 8, and remained lower on day 13, 15-20, and 22 (Figure 9). 31-day mean and median glucose levels were reduced −15.0% (range: −34.3 to 0.7%; *p* = 0.0057) and –15.2% (range: −33.2% to 0.4%; *p* = 0.0058) on LCHF treatment, respectively. 31-day time in range (70-110 mg/dL) increased 35.4% (range: 1.1 to 122.3%), standard deviation reduced −34.9% (range: −57.5 to 5.7%), and coefficient of variance reduced −25.4% (range: −41.0 to 12.4%) on LCHF. 31-day daily glucose minimum and maximums were also reduced on LCHF. 31-day median glucose was significantly lower throughout fasting (00:00 to 07:00) and feeding (07:01 to 23:59) windows (*p* = *0.004;* Figure 10). Interestingly, 30% of subjects had 31-day mean and median glucose (mean glucose range: 111.7-115.2 mg/dL; Figure 9) and fasting glucose > 100 mg/dL (Figure 10) throughout HCLF treatment, which is consistent with prediabetes glycemic values (62). These subjects were also the greatest responders to carbohydrate restriction (range: −21.5 to −34.3% mean glucose). When observing whether there was a significant relationship between the mean glucose while on HCLF versus the percentage change in mean glucose between LCHF and HCLF, we observed a significant (*p* = 0.0077) interaction with a large effect size (*r*^2^ = 0.6094) indicating that those individuals with a higher mean glucose, are more responsive to carbohydrate restriction treatment (Figure 9). Importantly, 30% of subjects who had a 31-day average mean, median, and fasting glucose > 100 mg/dL on HCLF (range: 111.68-115.19 mg/dL), were also the largest glycemic responders to carbohydrate restriction and also reported the highest fat oxidation rates during LCHF, with a large inverse relationship (*p* = 0.0069; *r*^2^ = 0.6194) across the entire cohort between the percent change in mean glucose when switching to LCHF and the peak fat oxidation rate at 86.4% VO_2max_ indicating that those individuals with the greatest change in glycemic control also had the greatest shift in global metrics of systemic metabolic adaptation to diet. To explore whether peak fat oxidation rates on LCHF were associated with circulating lipids, we found that higher peak fat oxidation (*p* = 0.0034; *r*^2^ = 0.6775) predicted higher total cholesterol, with trends for triglycerides (*p* = 0.0730; *r*^2^ = 0.3474; X = Peak Fat Oxidation on LCHF; Y = Triglycerides on LCHF; Y = 29.06*X + 24.62), suggesting a potential relationship between changes in fat oxidation rates and circulating lipid metabolism. These findings in the 30% of subjects with pre-diabetes in our study could not be explained by underlying demographic, body composition or running experience as these subjects with glycemic values consistent with pre-diabetes (62) had near equivalent age (pre-diabetic: 41.67 y/o; cohort: 39.3 y/o), running experience (pre-diabetic: 8.67 y; cohort: 9.70 y), lower weight (pre-diabetic: 84.03 kg; cohort: 86.70 kg), BMI (pre-diabetic: 25.37 kg/m^2^; cohort: 26.2 kg/m^2^), body fat [% (pre-diabetic: 14.8%; cohort: 15.7%) & kg (pre-diabetic: 12.7 kg; cohort: 14.1 kg)], and relative VO_2max_ (pre-diabetic: 60.97 mL/kg/min; cohort: 58.70 mL/kg/min). Additionally, this prediabetic phenotype was present in these subjects despite them losing weight on both nutritional strategies (LCHF: –2.3 ± 1.3 kg; HCLF: –2.1 ± 3.2 kg).

## Discussion

There are four key findings of this study (Figure 1). (i) Athletes achieved equivalent exercise performances during a 1,609 m time trial and a 6 × 800 m interval session after a 31-day habituation to LCHF or HCLF diets when controlling calories, training load, and body composition changes across groups. (ii) During the latter stages of the 6 × 800m interval session, athletes achieved the highest rates of fat oxidation yet reported. According to current understanding, this is paradoxical since these high rates were measured in subjects exercising at an intensity (86.4 ± 6.24% VO_2max_) at which the rate of fat oxidation should be approaching zero (25, 26, 30), not increasing. (iii) 31-days on each diet produced equivalent fasting insulin, hsCRP, and HbA_1c_, with elevated total, low-density lipoprotein, and high-density cholesterol on LCHF. (iv) LCHF consistently reduced glucose levels and variability with a large inverse relationship observed between mean glucose on HCLF and the percent change in mean glucose when switching to LCHF. Importantly, 30% of subjects who had a 31-day mean, median, and fasting glucose > 100 mg/dL on HCLF were also the largest responders (i.e., glycemic change over diet and peak fat oxidation rates) to carbohydrate restriction. No subjects on LCHF had a 31-day average mean glucose > 100 mg/dL. Additionally, relationships were observed between glycemic change, peak fat oxidation, and circulating lipids, as the larger the reduction in mean glucose on LCHF the larger the peak fat oxidation on LCHF, and the larger the peak fat oxidation on LCHF and the higher circulating lipids were. These results challenge the existing paradigm that diets with higher carbohydrate intake are superior for athletic performance, even during shorter-duration, higher-intensity exercise. Critically, these results demonstrate that lower carbohydrate intake may be a therapeutic strategy, even in athletes, to improve glycemic control, particularly in those with, or at risk for diabetes, without requiring changes in body composition or physical activity. Interestingly, these results also demonstrate a unique association between glycemic responsiveness to carbohydrate restriction, fat oxidation rates, and circulating lipids, suggesting an important relationship between continuous glycemic parameters and systemic metabolic responsiveness.

### Unaltered athletic performance during the 1,609 meter time trial and during the interval session of 6 × 800 m

Performance during the 1,609 m time trials was the same when athletes ate HCLF or LCHF diets. This is in keeping with our previous study (31) in which the 5-km time trial performances of athletes, similar in ability to those studied here, were equivalent on either diet. It adds further weight to the conclusions from two recent meta-analyses (63, 64) that that LCHF and HCLF diets produce equivalent performances across a wide range of athletic events.

Further, to search for a performance difference between dietary interventions, we asked the athletes to perform an interval session involving six repetitions of 800m at a pace equivalent to an exercise intensity of ∼85% VO_2max_. Our reasoning was that if the pre-exercise muscle glycogen stores are a critical determinant of exercise performance and if the LCHF diet is associated with lower muscle glycogen concentrations in recreational athletes (28) (but perhaps not in highly competitive athletes (65)), and since very high rates of muscle glycogen use are measured during 800m repetitions (66) so that, if significant muscle glycogen depletion can be produced by a high intensity interval session, then any impaired performance of athletes eating the LCHF diet should become apparent in the latter intervals of that session.

For example, Impey et al. reported rates of muscle glycogen use of 12.7 and 7.0 mmol/min in the gastrocnemius and vastus lateralis muscles respectively of recreational male athletes performing 800m repetitions at 100% VO_2max_ (66). Webster et al. reported that pre-exercise vastus lateralis glycogen concentrations were 85 mmol/kg in well-trained recreational athletes eating the LCHF diet (28). Whilst appreciating that rates of muscle glycogen use are reduced in those eating the LCHF diet (28, 65), according to these data a starting muscle glycogen concentration of 85 mmol/kg in the vastus lateralis muscle would be depleted after just 12 minutes of high intensity exercise. Our athletes exercised for ∼21 minutes during the 6 × 800m repetitions; sufficient time to produce substantial glycogen depletion.

In contrast to our expectation, based on this prediction that significant muscle glycogen depletion would occur in athletes following the LCHF diet and this would impair their performance, in fact exercise performance was identical across all the intervals on either diet (Tables 4, 5; Figure 4 and Supplementary Table 1).

These finding raise the important question of why our two studies have failed to detect diet-induced differences in performance whereas prior meticulously conducted studies (16–18) detected meaningful differences in their studies of Olympic standard race walkers. Five key factors may have contributed to these differences: randomization, dietary controls during exercise, training load, body composition, and dietary habituation timeline. Prior studies with differing results allowed subjects to choose the diet they preferred (16–18). In addition, during the final performance trial involving simulated races of up to 25-km, subjects on the LCHF diet were provided with carbohydrate-free “non-caloric fluid (water or artificially sweetened drinks)” (16) and “LCHF cookies” (17) whereas subjects on the HCLF diet received “sports drink, sports gels and confectionary” providing “∼60g CHO” every hour (16, 17). As a result, blood glucose levels were lower in the LCHF group in the two trials (16, 17) in which it was measured, with a trend toward a progressive hypoglycemia in one trial [figure 5A from (17)]. As the authors of those studies appreciate, even in the absence of hypoglycemia, carbohydrate ingestion alone can have an ergogenic effect even if the carbohydrate is not ingested (67). Thus, these trials did not control for the potential effects of carbohydrate ingestion during exercise. The potential role of hypoglycemia in explaining differences in exercise performance has recently been revisited (29). The intensified training load and across group differences in body composition in these trials (16–18) also illustrates key differences as increased physical activity levels (68) and body weight reductions (69) both illustrate biological stressors requiring adaptation and may independently impact performance. The increased physical activity across groups and more significant reductions in bodyweight in LCHF arm (16–18), on top of introducing a diet which requires systemic metabolic reprogramming (70), illustrate three co-administered biological stressors all requiring adaptions and which may influence performance. Lastly, our analyses allowed for a 4-week adaptation timeline, which is ≥ 33% longer than prior trials describing negative performance impacts (16–18). Thus, it is not surprising that when we controlled randomization (within-subject), dietary controls during performance testing, calories, training load, and body compositional changes across groups to allow for the isolation of diet-induced changes across these key parameters, we observed different results from prior observations (16–18). Of note, when Burke et al. (16) attempted to repeat their findings (18), they did not detect differences between LCHF and HCLF in real world race performance via IAAF points [figure 5C from; (16)], a point acknowledged by the authors in the abstract.

### Subjects achieved amongst the highest rates of fat oxidation yet measured during the latter stages of the interval session when eating the LCHF diet

The described method for measuring maximal rates of fat oxidation during exercise is to have subjects exercise for short periods of approximately 3 minutes at exercise intensities that gradually increase (22–24). Using this method, the highest rates of fat oxidation are generally achieved at submaximal exercise intensities of between 55-72% VO_2max_. Maximal rates of fat oxidation measured with this method are usually in the range of 0.5-0.6 g/min. Importantly, at higher exercise intensities rates of fat oxidation fall precipitously, reported reaching 0% at exercise intensities > 85% VO_2max_.

Higher rates of fat oxidation have been measured in athletes adapting to the LCHF diet. Volek et al. measured rates of 1.2 g/min in elite ultra-marathon runners performing prolonged exercise (180 minutes) at 64% VO_2max_ with peak fat oxidation of 1.54 g/min at 70.3% VO_2max_ (65). Webster et al. measured identical values in well-trained recreational athletes during 120 minutes’ exercise at 55% of peak power output (28); whereas Burke et al. measured rates in excess of 1.4-1.5 g/min during the final km of 25-km time trials in Olympic class race walkers (16, 17). In a case study of an elite Ironman triathlete, Webster et al. reported a peak fat oxidation rate of 1.6 g/min in a single cyclist for the full duration of a 100-km cycle trial performed at an average power output of 224 W (28, 71). Shaw et al. demonstrate fat oxidation ranging from 0.88 to 1.51 g/min following 31-day of LCHF habituation (72). Our data is in line with these prior studies showing elevated fat oxidation rates (LCHF: 1.58 ± 0.33 g/min).

Thus, the values for fat oxidation rates measured in these recreational athletes are unusually high, particularly when they were measured under experimental conditions expected to produce values close to 0g/min. However, what is particularly unique in our findings is that we observed the peak fat oxidation rates (LCHF: 1.58 ± 0.33 g/min) at 86.40 ± 6.24% VO_2max_. Additionally, 30% of subjects reported record high fat oxidation rates > 1.85 g/min (1.95 ± 0.08 g/min), ranging from 1.86 to 2.01 g/min, which to our knowledge is the highest rates ever recorded. These unique findings may be a result of subject age and athletic status and/or due to the unique controls incorporated into this study to isolate diet-induced effects (i.e., randomization, calories, training load, and body composition changes controlled across groups).

### Cardiometabolic impact of LCHF and HCLF

Three out of eight cardiometabolic markers were significantly modulated by diet, most notably post-diet LCHF vs. HCLF total cholesterol (238 vs. 208 mg/dL, 14%; *p* = 0.007) and HDL-C (68 vs. 57 mg/dL, 20%; *p* = 0.045) concentrations. Although the overall LDL-C condition effect was significant (*p* = 0.03), the LCHF diet did not increase LDL-C concentrations beyond HCLF after four-weeks (155 vs. 137 mg/dL; 18% *p* = 0.30), implying that post-diet hypercholesterolemia effects were predominantly determined by changes in HDL-C fraction. We anticipated based on our prior work (73) and others (65, 74, 75) that competitive runners would experience significant blood lipid changes within weeks after starting a LCHF designed to induce nutritional ketosis (≥ 0.5 mM *R*-βHB). The significant main effects in this study were directly attributable to the between-diet differences in dietary fat and fat composition, however, the fact that more than half of participants had borderline elevated total cholesterol, LDL-C, and HDL-C at baseline was somewhat unexpected. While carryover effects were ruled out by identical concentrations at baseline (i.e., both conditions started the same), this effect may have indirectly revealed that a peak or plateau had not been reached over our four-week intervention, making blood lipid trends harder to speculate. Moreover, it is unclear if similar dietary interventions in mildly hypercholesterolemic athletes will exert any significant impact on cardiometabolic indices that are beyond the effects induced by their habitual diet. Individual cardiometabolic responses are available for review in the supplement (Supplementary Figure 1).

We detected a small, but significant change in weight over time, primarily derived from fat mass. Despite energy intake and training load being similar between LCHF and HCLF diets, weight-loss and diet did not modulate triglycerides, insulin/HbA_1c_, and inflammation (hsCRP). Based on prior evidence (73, 76) the LCHF diet was projected to lower cardiometabolic markers beyond a HCLF diet, even in the absence of weight-loss (77); however, we did not observe these results with our between-diet isocaloric feeding design. Additionally, it is important to acknowledge that HbA_1c_ is a 2–3-month biomarker that we quantified to predict directional trends rather than significant changes over four weeks. Based on our findings, we expect that these markers will continue decreasing after four-weeks of LCHF, similar to isocaloric HCLF feeding when duration, energy intake, and weight are controlled between conditions.

### Improved glycemic control when eating the LCHF diet

Continuous glucose monitors are unique tools which allow researchers to extract changes in glycemic control every ≤ 15 min over extended periods of time without relying on the limitations at a single timepoint which may only provide limited biological feedback (78, 79). Importantly, (i) CGM tracks long-term with HbA_1*c*_ (54–56), (ii) shorter term CGM readings (10-14d) are good estimates of 3-month CGM averages (57), and (iii) can also capture both fasting and post-prandial differences in glucose (validated diagnostic tool) demonstrating a powerful monitoring tool for glycemic control, particularly in interventions not long enough to confirm alterations in HbA_1*c*_ (< 2-3 months in length). The value of these tools has been shown in previous observations utilizing lower carbohydrate (80) or ketogenic interventions (81) where continuous glucose patterns found key shifts in glycemic control before and/or in the absence of changes in traditional biochemical cardiometabolic biomarkers. When measuring continuous glucose levels every 15 min over a 31-day period, we observed improvements across all glycemic parameters in virtually all subjects on the LCHF diet, with initial significant differences in mean glucose observed on day 8 of dietary habituation. Carbohydrate restriction is a known therapeutic strategy to help facilitate improvements in glycemic control and other key metabolic parameters in other clinical conditions such as obesity (82), type-1 diabetes (36), and type-2 diabetes (33, 35). The improved glycemic mean, median, and variability on the LCHF diet in middle-aged athletes was observed in a rigorous, randomized cross-over study design without the confounding influence of caloric, training load/physical activity, and body composition differences across diets. These illustrate critical controls allowing us to extract diet-induced impact on glycemic parameters as prior observations have shown that caloric intake and changes in body weight both influence glucose levels regardless of diet (83, 84). Additionally, prior evaluations have found that intensified training programs can disrupt not only glycaemia and mitochondrial function, but also performance (68).

While there have been small short-term investigations exploring glycemic control during exercise in athletes (79, 85), very few studies have investigated the relationship between the long-term (i.e., ≥ 1 month) 24-h glycemic control while also observing performance. Nolan et al. reported the impact of a ketogenic diet on an individual type-1 diabetic cyclist during a 20-day, 4011-km race (86). This case report demonstrated remarkable glycemic control for a Type-1 Diabetic compared to historical glycemic norms for Type-1 Diabetics during this 20d race window, but nature of the report did not allow for the comparison of performance on- and off-diet.

While all subjects were “healthy,” with normal bodyweight and BMI, and competitive middle-aged athletes without any medical diagnoses, we observed that when continuously monitoring glucose parameters over a 31-day period, 30% of subjects on a HCLF diet had mean, median, and fasting BG > 100 mg/dL, consistent with pre-diabetes interstitial glucose values using analogous technology (62). This is consistent with a prior analysis which found that 30% of sub-elite endurance athletes exercising > 6-hour per week had undetected pre-diabetes when measured via continuous glucose monitoring devices (87). These subjects fitting the pre-diabetes glycemic phenotype in our study could not be explained by underlying demographics, body composition or physical activity differences as these pre-diabetic subjects had near equivalent age (pre-diabetic: 41.67 y/o; cohort: 39.3 y/o), running experience (pre-diabetic: 8.67 y; cohort: 9.70 y), lower weight (pre-diabetic: 84.03 kg; cohort: 86.70 kg), BMI (pre-diabetic: 25.37 kg/m2; cohort: 26.2 kg/m^2^) and body fat [% (pre-diabetic: 14.8%; cohort: 15.7%) & kg (pre-diabetic: 12.7 kg; cohort: 14.1 kg)], and higher VO_2max_ (pre-diabetic: 60.97 mL/kg/min; cohort: 58.70 mL/kg/min) when compared to the entire cohort. This is in line with the understanding that multiple factors contribute to diabetes onset (88, 89), some of which may go undetected until overt diagnosis. Potential explanations for early pathogenic progression of diabetic dysglycemia include genetic predisposition, adiposity-induced insulin resistance, fasting insulin, and beta-cell dysfunction (88). However, markers of elevated adiposity were not higher in the prediabetic group. In fact, this sub-cohort lost weight on both dietary protocols. Additionally, circulating lipids tended to be lower on the HCLF diet suggesting lipids could not explain dysglycemia on HCLF. Fasting insulin was not different across diet groups, nor were baseline or post-HCLF diet fasting insulin levels associated with elevated mean glucose on HCLF (*p* = 0.4048). However, this does not exclude the possibility that dynamic changes in post-prandial insulin or beta-cell function couldn’t be contributing to this effect which we did not measure herein. While intense exercise overtraining has also been demonstrated to acutely disrupt mitochondrial and glycemic function (68), this dysfunction was reversed following reduction in activity and cannot explain our results as our subjects did not increase or decrease physical activity levels. While genetic predisposition may explain why 30% of healthy, active and normal weight individuals had pre-diabetes on HCLF, it cannot explain across treatment effects as the crossover design controlled for this variable. Our results demonstrate that all these subjects were able to reduce their mean and median glucose < 100 mg/dL on LCHF diet. While prior observations have demonstrated the ability to improve glycemic control in individuals with established pre-diabetes and obesity in the absence of exercise consumed a low-carbohydrate diet (< 100 g carbohydrates/day) (62), to our knowledge, this is the first observation to demonstrate the ability to detect and resolve pre-diabetes without changes in activity or changes across groups in body composition using carbohydrate restriction in competitive middle aged athletes. Importantly, Al-Ozairi et al. found that a 6-day LCHF diet in Type-2 Diabetic subjects who kept calories and bodyweight controlled were unable to find differences in mean and post-prandial glycaemia utilizing CGM devices (90). This could be due to the short treatment duration as we observed significant differences on day 8 of the isocaloric HCLF and LCHF diets. Alternatively, it may be explained by the influence of engaging in physical exercise regularly as Moholdt et al. found a 5% reduction in mean glucose levels using CGM without changes in bodyweight following a 5-day lower carbohydrate diet (i.e., 15% kcal carbohydrates) and exercise in obese subjects who were of similar age to our cohort (80). Our larger reduction in mean (15%) and median glucose (15.2%) compared to Moholdt’s (5%) is likely explained by Moholdt’s higher percentage of carbohydrate (15%), shorter duration of diet (5-days), and different metabolic phenotype in their cohort (i.e., sedentary; BMI > 30 kg/m^2^). Our observation of consistent improvements in glycaemia in middle-aged athletes (30% pre-diabetic glycemic phenotype), without impairing performance, illustrates a promising therapeutic strategy for improving glycemic control, without requiring body composition and physical activity change.

### Higher mean glucose levels predict glycemic response to carbohydrate restriction, glycemic response to carbohydrate restriction predicts peak fat oxidation, and peak fat oxidation predicts circulating lipids

Our study found that 30% of subjects who had a 31-day average mean and median glucose > 100 mg/dL on HCLF (range: 111.68 – 115.19 mg/dL; pre-diabetic phenotype), were also the largest glycemic responders to carbohydrate restriction. Importantly, when looking to observe if the entire cohort also observed a relationship between 31-day average mean glucose on HCLF diet and percentage change in mean glucose between LCHF and HCLF diet, we observed a large significant inverse relationship, indicating that those individuals with a higher mean glucose, are more responsive to carbohydrate restriction treatment, not just those with pre-diabetic glycemic phenotypes. As our study and prior literature suggests this change is in response to diet and not other factors (i.e., calories, body composition, altered activity levels, cardiometabolic factors), thus indicating that as individuals develop increasing levels of mean BG, they may become more responsive to therapeutic carbohydrate restriction. Interestingly, the 30% of subjects who had a 31-day average mean and median glucose > 100 mg/dL on HCLF (range: 111.68 – 115.19 mg/dL; pre-diabetic phenotype), also reported the highest fat oxidation rates during LCHF, with a large inverse relationship (*r*^2^ = 0.6194; *p* = 0.0069) across the entire cohort between the percent change in mean glucose when switching to LCHF and the peak fat oxidation rate at 86.4% VO_2_max indicating that those individuals with the greatest change in glycemic control also had the greatest shift in global metrics of systemic metabolic adaptation to diet. While multiple studies have shown reductions in glucose (35, 80, 83) and elevations in fat oxidation (16–18, 28, 31, 91) on a LCHF diet, we are unaware of any data which has demonstrated that the magnitude of glycemic changes across diet predicted the magnitude of peak fat oxidation rates. Interestingly, we also observed that higher peak fat oxidation levels on LCHF predicted higher total cholesterol on LCHF suggesting a potential interaction between higher rates of fat turnover and higher levels of circulating lipids while on a diet that restricts carbohydrates and increases fat intake. While elevated fat oxidation rates have been observed on LCHF diet in the absence in changes of insulin or calories, explained by elevated fat intake, (91), they did not see a change in glucose levels nor did they explore whether the magnitude of fat oxidation rate was associated with glucose or lipid parameters. In line with our data, there has been a report demonstrating that individuals with healthy bodyweight undergoing a LCHF diet can have elevated circulating lipid (i.e., LDL-C) (92). While this prior observation did not look at either total cholesterol or fat oxidation rates, in light of our data, there remains a possibility that these individuals (92), have elevated levels of systemic fat oxidation which requires further analyses. However, in disagreement with these findings we did not observe a relationship between fat oxidation and LDL, a relationship between baseline Trig/HDL predict LDL-C, nor any LDL-C levels “hyper-responders” (LDL-C: > 200 mg/dL) which indicates our findings may not translate to this unique cohort (92). The ability for (i) 31-d mean glucose on HCLF to predict changes in mean glucose following carbohydrate restriction, (ii) changes in mean glucose with carbohydrate restriction to predict peak oxidation rates, and (iii) peak fat oxidation to predict total cholesterol suggests a unique predictable physiologic relationship between glycemia, substrate oxidation, and circulating lipids biomarkers which requires further validation.

## Limitations

This study had middle-aged competitive male athletes which may limit our understanding of the translatability of these findings to female athletes due to potential differences across sex on the magnitude of metabolic response (93–95), particularly for those women in middle age during pre-menopause and post-menopause who may benefit most due to elevated risk for cardiovascular and metabolic disease (96, 97). While our short-duration high-intensity exercise (6 × 800 m) would be sufficient to reduce muscle glycogen content based on prior work, (28, 65, 66) we did not measure muscle glycogen content so we cannot say for certain what levels of muscle glycogen were achieved and if they were associate with elevated fat oxidation levels during exercise. While HbA_1c_ is gold-standard for diagnosing diabetic phenotype due to its established role in diabetes, our dietary intervention was 4 weeks in length, an insufficient time to observe the full diet-induced impact on HbA_1c_ which requires a minimum of 8-12 weeks (98, 99). We utilized CGM to capture the 4-week 24-h glycemic control as (i) CGM tracks long-term to HbA_1c_ (54–56), (ii) shorter term CGM readings (10-14d) are good estimates of 3-month CGM averages (57), and (iii) can also capture both fasting and post-prandial differences in glucose which is a validated diagnostic tool (Figures 9, 10). Although limitation have been cited when looking different CGM technology and different insertion sites, both technology and insertion site were controlled in our analyses (100). However, it is important to note the clear limitation of HbA_1c_ and oral glucose tolerance test (OGTT) in our present analyses and why CGM was the primary glycemic metric. It is well-established that for a given HbA_1c_ value, there is a wide-range of mean glucose concentrations, and for any given mean glucose concentration, there is a wide-range of HbA_1c_ values, suggesting some limitation around this biomarker (101). Thus, some expert consensus has argued for moving beyond just HbA_1c_ at the individual levels (102). Additionally, it has been known for decades that OGTT is inappropriate for individuals not adhering to an HCLF diet as this test was only validated under high-carbohydrate consumption (103). While we feel confident that our 24-h 4-week glycemic values across subjects accurately capture the glycemic impact over our study duration, future studies with benefit from longer dietary interventions 2-3 m in duration to capture changes in HbA_1c_.

## Conclusion

We demonstrated that a habituating to a LCHF for ≥ 4weeks in 30 to 50-years old competitive male athletes resulted in equivalent short duration, high-intensity performance without differences in calories, training load, and body composition across groups. We observed record high peak oxidation rates with elevations in cholesterol in LCHF. Interestingly, we found a 30% incidence of pre-diabetic glycemic phenotype in seemingly healthy athletes consuming a high carbohydrate diet without detectable risk factors for pre-diabetes. All individuals experienced reductions in 31-day average glucose means, median, and variability with carbohydrate restriction (LCHF) which resolved the pre-diabetic phenotype across all subjects without requiring caloric restriction, increased physical activity, or significant changes in body composition across groups. Interestingly, the average glucose during high carbohydrate consumption predicted the degree of glycemic response to carbohydrate restriction suggesting that individuals with higher starting glucose may benefit most from carbohydrate restriction. Surprisingly, we also found that the magnitude of glucose reduction during carbohydrate restriction predicted the elevation in fat oxidation rates during exercise suggesting that glucose response is linked to systemic fat oxidation. Taken together, LCHF may represent a therapeutic strategy to improve glucose levels, particularly in those at risk for diabetes, without compromising high intensity exercise performance in middle-aged athletes. Future studies should evaluate the impact of these dietary strategies in middle-aged women who are at elevated risk for cardiovascular and metabolic disease.

## Data availability statement

The original contributions presented in this study are included in this article/Supplementary material, further inquiries can be directed to the corresponding authors.

## Ethics statement

The studies involving human participants were reviewed and approved by Institutional Review Board of Grove City College (IRB number 110-2021). The patients/participants provided their written informed consent to participate in this study.

## Author contributions

PP and TN conceived the original study design. PP, JB, DJ, NT, HG, AJ, KJ, and DD’A conducted the participant testing and collected all the data. KH designed the diets and provided the nutritional counseling. PP, AB, and AK conducted the data analysis. PP, TN, AK, and AB drafted the final manuscript. All authors have read and agreed to the published version of the manuscript.
