# Supplementary material for: Low and high carbohydrate isocaloric diets on performance, fat oxidation, glucose and cardiometabolic health in middle age males
Source: Front Nutr. 2023 Feb 9;10:1084021. doi: 10.3389/fnut.2023.1084021 (PMC9946985; doi:10.3389/fnut.2023.1084021)
Supplement: Supplementary file 1 [file Data_Sheet_1.PDF]

Table S1. Physiological, Metabolic, Respiratory, Perceptual, and Performance Data Collected During the Repeated Sprint Protocol (*n* = 10)

		Time							
		**Set 1**	**Set 2**	**Set 3**	**Set 4**	**Set 5**	**Set 6**	***P-Value Interactions***	***P-Value Main Effects***
					**VO_2 (L/min)**				
**LCHF**	**Pre**	3.64 ± 0.34	3.91 ± 0.48	3.84 ± 0.49	3.92 ± 0.33	3.82 ± 0.54	4.03 ± 0.53	Condition x Time, *P* = 0.248; η2p = 0.096<br>Condition x Set, *P* = 0.409; η2p = 0.103<br>Time x Set, *P* = 0.458; η2p = 0.096<br>Condition x Time x Set, *P* = 0.158; η2p = 0.158	Condition, *P* = 0.423; η2p = 0.073<br>Time, *P* = 0.534; η2p = 0.044<br>Set, *P* < **0.001**; η2p = 0.455
	**Post**	3.91 ± 0.43	4.02 ± 0.40	4.02 ± 0.44	3.92 ± 0.37	3.90 ± 0.46	4.12 ± 0.47		
**HCLF**	**Pre**	3.71 ± 0.54	3.88 ± 0.45	3.83 ± 0.47	3.78 ± 0.51	3.92 ± 0.57	3.99 ± 0.54		
	**Post**	3.73 ± 0.53	3.70 ± 0.49	3.73 ± 0.69	3.86 ± 0.51	3.88 ± 0.45	3.98 ± 0.55		
					**VCO_2 (L/min)**				
**LCHF**	**Pre**	3.24 ± 0.37	3.39 ± 0.47	3.32 ± 0.44	3.36 ± 0.38	3.23 ± 0.52	3.43 ± 0.54	Condition x Time, *P* = 0.576; η2p = 0.036<br>Condition x Set, *P* = 0.077; η2p = 0.192<br>Time x Set, *P* = 0.085; η2p = 0.188<br>Condition x Time x Set, *P* = 0.351; η2p = 0.113	Condition, *P* = 0.091; η2p = 0.258<br>Time, *P* = 0.423; η2p = 0.073<br>Set, *P* = 0.133; η2p = 0.166
	**Post**	3.26 ± 0.40	3.25 ± 0.38	3.27 ± 0.38	3.10 ± 0.37	3.09 ± 0.36	3.25 ± 0.43		
**HCLF**	**Pre**	3.58 ± 0.63	3.57 ± 0.47	3.43 ± 0.52	3.48 ± 0.60	3.48 ± 0.67	3.63 ± 0.64		
	**Post**	3.63 ± 0.46	3.33 ± 0.40	3.44 ± 0.56	3.48 ± 0.47	3.47 ± 0.41	3.61 ± 0.49		
					**RER**				
**LCHF**	**Pre**	0.90 ± 0.08	0.87 ± 0.56	0.86 ± 0.58	0.86 ± 0.53	0.87 ± 0.05	0.85 ± 0.05	Condition x Time, *P* = 0.010; η2p = 0.536<br>Condition x Set, *P* = 0.011; η2p = 0.274<br>Time x Set, *P* = 0.377; η2p = 0.108<br>Condition x Time x Set, *P* = 0.269; η2p = 0.129	Condition, *P* = 0.001; η2p = 0.713<br>Time, *P* = 0.005; η2p = 0.601<br>Set, *P*<0.001; η2p = 0.540
	**Post**	0.83 ± 0.05$^{*\#}$	0.80 ± 0.03$^{*\#}$	0.81 ± 0.04$^{*\#}$	0.79 ± 0.04$^{*\#}$	0.79 ± 0.06$^{*\#}$	0.78 ± 0.03$^{*\#}$		
**HCLF**	**Pre**	0.95 ± 0.06	0.92 ± 0.05	0.90 ± 0.05	0.90 ± 0.04	0.87 ± 0.07	0.89 ± 0.07		
	**Post**	0.97 ± 0.06	0.90 ± 0.04	0.91 ± 0.03	0.90 ± 0.04	0.89 ± 0.03	0.90 ± 0.02		
					**VO_2 (ml/kg/min)**				
**LCHF**	**Pre**	43.4 ± 5.8	46.4 ± 6.6	45.5 ± 6.6	46.1 ± 5.6	45.2 ± 6.1	48.2 ± 6.0	Condition x Time, *P* = 0.070; η2p = 0.320<br>Condition x Set, *P* = 0.397; η2p = 0.105<br>Time x Set, *P* = 0.452; η2p = 0.096<br>Condition x Time x Set, *P* = 0.203; η2p = 0.144	Condition, *P* = 0.457; η2p = 0.063<br>Time, *P* = 0.263; η2p = 0.136<br>Set, *P* < 0.001; η2p = 0.501
	**Post**	48.0 ± 4.8	49.4 ± 4.5	49.3 ± 4.3	47.9 ± 4.7	48.2 ± 5.5	50.4 ± 2.2		
**HCLF**	**Pre**	44.5 ± 7.7	46.9 ± 8.4	46.1 ± 7.9	45.5 ± 7.4	47.2 ± 9.3	47.9 ± 8.2		
	**Post**	44.8 ± 5.1	44.7 ± 5.7	45.2 ± 6.2	46.5 ± 5.2	46.8 ± 5.6	48.2 ± 4.9		
					**Ve (L/min)**				
**LCHF**	**Pre**	97.3 ± 12.9	107.3 ± 12.5	106.8 ± 11.8	109.7 ± 10.5	107.3 ± 11.4	113.5 ± 11.9	Condition x Time, *P* = 0.999; η2p = 0.000<br>Condition x Set, *P* = 0.357; η2p = 0.112<br>Time x Set, *P* = 0.306; η2p = 0.121<br>Condition x Time x Set, *P* = 0.538; η2p = 0.084	Condition, *P* = 0.956; η2p = 0.000<br>Time, *P* = 0.925; η2p = 0.001<br>Set, *P* < 0.001; η2p = 0.622
	**Post**	99.5 ± 13.2	106.4 ± 12.6	108.6 ± 13.1	106.9 ± 12.1	105.5 ± 11.8	113.8 ± 13.4		
**HCLF**	**Pre**	99.3 ± 15.1	107.3 ± 12.0	106.5 ± 14.1	106.3 ± 12.5	109.3 ± 13.9	112.2 ± 14.2		
	**Post**	102.3 ± 12.8	102.6 ± 10.3	105.3 ± 13.1	108.0 ± 10.6	108.4 ± 10.4	113.1 ± 12.4		
					**RR (breath/min)**				
**LCHF**	**Pre**	37.3 ± 8.1	40.5 ± 7.4	41.4 ± 6.1	41.8 ± 7.1	40.6 ± 9.8	43.3 ± 8.2	Condition x Time, *P* = 0.273; η2p = 0.132<br>Condition x Set, *P* = 0.409; η2p = 0.103<br>Time x Set, *P* = 0.287; η2p = 0.125<br>Condition x Time x Set, *P* = 0.784; η2p = 0.051	Condition, *P* = 0.806; η2p = 0.007<br>Time, *P* = 0.622; η2p = 0.028<br>Set, *P* < 0.001; η2p = 0.759
	**Post**	36.7 ± 7.8	38.5 ± 7.3	39.6 ± 7.0	40.7 ± 6.3	39.9 ± 6.9	42.9 ± 7.3		
**HCLF**	**Pre**	36.4 ± 7.6	39.5 ± 7.5	40.5 ± 8.6	40.6 ± 7.8	42.0 ± 7.5	42.2 ± 8.5		
	**Post**	38.1 ± 7.1	38.8 ± 7.1	40.7 ± 7.0	41.8 ± 8.1	41.4 ± 7.1	42.9 ± 8.5		
					**HR (beats/min)**				

**LCHF**	**Pre**	152.6 ± 11.7	160.2 ± 9.1	161.0 ± 9.8	160.6 ± 9.2	162.4 ± 9.6	165.8 ± 10.0	Condition x Time, *P* = 0.124; η2p = 0.243<br>Condition x Set, *P* = 0.150; η2p = 0.160<br>Time x Set, *P* = 0.240; η2p = 0.135<br>Condition x Time x Set, *P* = 0.732; η2p = 0.058	Condition, *P* = 0.698; η2p = 0.017<br>Time, *P* = 0.935; η2p = 0.001<br>Set, *P*<0.001; η2p = 0.704
	**Post**	158.3 ± 14.5	160.8 ± 13.4	163.3 ± 11.5	162.8 ± 13.8	164.5 ± 15.5	168.9 ± 13.9		
**HCLF**	**Pre**	157.9 ± 8.7	160.5 ± 7.2	160.4 ± 8.0	162.0 ± 7.2	165.1 ± 9.2	166.0 ± 8.2		
	**Post**	155.8 ± 9.4	156.5 ± 8.9	159.7 ± 9.0	160.2 ± 8.2	161.9 ± 8.0	163.1 ± 11.1		
**Carbohydrate Oxidation (g/min)**									
**LCHF**	**Pre**	3.42 ± 1.32	3.19 ± 1.16	3.11 ± 1.17	3.13 ± 1.01	2.84 ± 1.03	3.01 ± 1.19	Condition x Time, *P* = 0.020; η2p = 0.467<br>Condition x Set, *P* = 0.014; η2p = 0.265<br>Time x Set, *P* = 0.037; η2p = 0.225<br>Condition x Time x Set, *P* = 0.232; η2p = 0.137	Condition, *P* = 0.004; η2p = 0.611<br>Time, *P* = 0.012; η2p = 0.521<br>Set, *P* < 0.001; η2p = 0.439
	**Post**	1.96 ± 1.18*#	1.56 ± 1.15*#	1.65 ± 1.06*#	1.08 ± 0.66*#	1.15 ± 0.73*#	1.26 ± 0.73*#		
**HCLF**	**Pre**	4.35 ± 1.46	3.81 ± 1.06	3.29 ± 1.10	3.54 ± 1.25	3.32 ± 1.40	3.54 ± 1.55		
	**Post**	4.54 ± 0.79	3.29 ± 0.62	3.66 ± 0.60	3.47 ± 0.73	3.34 ± 0.65	3.64 ± 0.61		
**Fat Oxidation (g/min)**									
**LCHF**	**Pre**	0.55 ± 0.45	0.72 ± 0.37	0.72 ± 0.39	0.77 ± 0.37	0.85 ± 0.34	0.85 ± 0.40	Condition x Time, *P* = 0.008; η2p = 0.561<br>Condition x Set, *P* = 0.335; η2p = 0.116<br>Time x Set, *P* = 0.265; η2p = 0.130<br>Condition x Time x Set, *P* = 0.504; η2p = 0.089	Condition, *P* = 0.002; η2p = 0.672<br>Time, *P* = 0.007; η2p = 0.567<br>Set, *P* < 0.001; η2p = 0.639
	**Post**	1.22 ± 0.48*#	1.42 ± 0.48*#	1.39 ± 0.51*#	1.50 ± 0.40*#	1.51 ± 0.49*#	1.57 ± 0.33*#		
**HCLF**	**Pre**	0.29 ± 0.31	0.52 ± 0.32	0.69 ± 0.32	0.60 ± 0.28	0.79 ± 0.40	0.68 ± 0.42		
	**Post**	0.23 ± 0.19	0.61 ± 0.29	0.49 ± 0.31	0.62 ± 0.23	0.68 ± 0.24	0.62 ± 0.21		
**RPE**									
**LCHF**	**Pre**	5.60 ± 0.96	6.30 ± 1.15	7.10 ± 0.99	7.60 ± 1.07	7.90 ± 1.10	8.40 ± 1.26	Condition x Time, *P* = 0.106; η2p = 0.263<br>Condition x Set, *P* = 0.693; η2p = 0.063<br>Time x Set, *P* = 0.303; η2p = 0.122<br>Condition x Time x Set, *P* = 0.158; η2p = 0.158	Condition, *P* = 0.909; η2p = 0.002<br>Time, *P* = 0.955; η2p = 0.000<br>Set, *P* < 0.001; η2p = 0.778
	**Post**	5.60 ± 1.42	6.30 ± 1.49	6.50 ± 0.97	7.20 ± 1.13	7.40 ± 1.42	8.30 ± 1.15		
**HCLF**	**Pre**	5.60 ± 1.34	6.40 ± 1.07	6.80 ± 1.13	6.90 ± 0.87	7.50 ± 0.84	8.30 ± 1.05		
	**Post**	5.40 ± 1.83	6.10 ± 0.87	6.90 ± 0.87	7.70 ± 0.67	8.10 ± 0.87	8.80 ± 0.91		
**Affect**									
**LCHF**	**Pre**	2.00 ± 1.88	0.70 ± 2.35	0.20 ± 2.61	-0.30 ± 2.62	-0.70 ± 2.98	-0.90 ± 3.17	Condition x Time, *P* = 0.516; η2p = 0.048<br>Condition x Set, *P* = 0.961; η2p = 0.022<br>Time x Set, *P* = 0.413; η2p = 0.102<br>Condition x Time x Set, *P* = 0.007; η2p = 0.289	Condition, *P* = 0.946; η2p = 0.001<br>Time, *P* = 0.385; η2p = 0.085<br>Set, *P* < 0.001; η2p = 0.687
	**Post**	1.60 ± 2.01	1.00 ± 2.44	0.40 ± 2.59	-0.30 ± 3.02	-0.40 ± 2.91	-1.10 ± 3.10		
**HCLF**	**Pre**	1.80 ± 1.93	1.10 ± 2.18	2.30 ± 2.40	0.10 ± 2.84	-0.40 ± 2.79	-0.90 ± 3.34		
	**Post**	1.50 ± 1.84	0.90 ± 2.13	0.00 ± 2.49	-0.50 ± 2.95	-0.60 ± 3.23	-1.30 ± 3.56		
**800m Split Time (sec)**									
**LCHF**	**Pre**	208.2 ± 18.1	207.1 ± 25.0	213.4 ± 18.9	214.4 ± 11.5	214.7 ± 12.6	209.2 ± 12.2	Condition x Time, *P* = 0.556; η2p = 0.040<br>Condition x Set, *P* = 0.124; η2p = 0.170<br>Time x Set, *P* = 0.377; η2p = 0.108<br>Condition x Time x Set, *P* = 0.990; η2p = 0.012	Condition, *P* = 0.695; η2p = 0.018<br>Time, *P* = 0.064; η2 p= 0.331<br>Set, *P* = 0.001; η2p = 0.367
	**Post**	201.0 ± 19.9	199.1 ± 13.0	207.6 ± 13.9	213.4 ± 13.3	210.8 ± 16.7	204.2 ± 13.3		
**HCLF**	**Pre**	201.9 ± 19.3	214.1 ± 18.7	212.6 ± 16.5	217.2 ± 16.7	212.3 ± 16.1	209.0 ± 18.6		
	**Post**	200.0 ± 21.7	206.9 ± 25.4	211.1 ± 17.6	216.1 ± 19.5	212.8 ± 14.6	207.1 ± 13.0		

Physiological, Metabolic, Respiratory, Heart Rate, and Perceptual Responses were assessed across six timepoints (*n* = 10). Values are Mean ± SD. Abbreviations: RPE-O = RPE for overall body; RPE = rating of perceived exertion (OMNI rating of exertion); RER = Respiratory exchange ratio; VO2 = oxygen consumption; VCO_2 = carbon dioxide production; VE = ventilation; RR = Respiratory Rate; LCHF = low carbohydrate high fat diet; HCLF= high carbohydrate low fat diet. * = $p < 0.05$ difference between diets; # = $p < 0.05$ difference from Set 1.

Table S2. Blood Metabolites Collected before and after the One-Mile Time Trial (n = 10)

		Time			
		**Pre-Ex**	**Post-Ex**	***P-Value Interactions***	***P-Value Main Effects***
		**Blood *R*-β-hydroxybutyrate (mmol/L)**			
**LCHF**	**Pre**	0.21 ± 0.11	0.19 ± 0.13	Condition x Time, ***P* = 0.001**; η2p = 0.711	Condition, ***P* = 0.005**; η2p = 0.611
	**Post**	0.67 ± 0.30*#†	0.53 ± 0.37*#†	Condition x Exercise, *P* = 0.279; η2p = 0.129	Time, ***P* = 0.005**; η2p = 0.598
**HCLF**	**Pre**	0.20 ± 0.12	0.19 ± 0.10	Time x Exercise, *P* = 0.291; η2p = 0.123	Exercise, *P* = 0.182; η2p = 0.189
	**Post**	0.18 ± 0.11	0.15 ± 0.05	Condition x Time x Exercise, *P* = 0.322; η2p = 0.109	
		**Blood Glucose (mg/dL)**			
**LCHF**	**Pre**	89.8 ± 9.1	132.0 ± 21.4#	Condition x Time, *P* = 0.118; η2p = 0.250	Condition, *P* = 0.481; η2p = 0.057
	**Post**	85.0 ± 10.2	119.6 ± 18.7#	Condition x Exercise, *P* = 0.576; η2p = 0.036	Time, *P* = 0.323; η2p = 0.108
**HCLF**	**Pre**	88.3 ± 10.8	127.8 ± 22.5#	Time x Exercise, *P* = 0.168; η2p = 0.200	Exercise, ***P*<0.001**; η2p = 0.879
	**Post**	93.1 ± 10.1	127.1 ± 18.3#	Condition x Time x Exercise, *P* = 0.738; η2p = 0.013	
		**Blood Lactate (mmol/L)**			
**LCHF**	**Pre**	1.14 ± 0.41	6.89 ± 2.77#	Condition x Time, *P* = 0.256; η2p = 0.141	Condition, *P* = 0.269; η2p = 0.134
	**Post**	1.15 ± 0.68	8.26 ± 2.65#	Condition x Exercise, *P* = 0.263; η2p = 0.137	Time, *P* = 0.279; η2p = 0.128
**HCLF**	**Pre**	1.24 ± 0.53	8.50 ± 2.49#	Time x Exercise, *P* = 0.179; η2p = 0.191	Exercise, ***P* < 0.001**; η2p = 0.939
	**Post**	1.35 ± 0.91	8.39 ± 1.76#	Condition x Time x Exercise, *P* = 0.350; η2p = 0.098	

Finger capillary blood glucose, *R*-β-hydroxybutyrate, and lactate were assessed across two timepoints (n = 10). Values are Mean ± SD. Abbreviations: Pre-ex = immediately before time trial; Post-ex = immediately after time trial; LCHF = low carbohydrate high fat diet; HCLF= high carbohydrate low fat diet. * = $p < 0.05$ difference between diets; # = $p < 0.05$ difference from Pre to Post-ex. † = significant interaction between LCHF and HCLF Post-diet ($p < 0.05$).

Table S3. Repeated Sprint Protocol Blood Metabolites (*n* = 10)

		Time								
		**Pre-Ex**	**Set 1**	**Set 2**	**Set 3**	**Set 4**	**Set 5**	**Post-Ex**	***P-Value* *Interactions***	***P-Value* *Main Effects***
		**Blood *R*-β-hydroxybutyrate (mmol/L)**								
**LCHF**	**Pre**	0.19 ± 0.12	0.13 ± 0.06	0.13 ± 0.06	0.15 ± 0.09	0.16 ± 0.09	0.17 ± 0.13	0.17 ± 0.12	Condition x Time, ***P* = 0.048**; η2p = 0.367	Condition, ***P* = 0.014**; η2p = 0.508
	**Post**	0.72 ± 0.73$^{\#\dagger}$	0.56 ± 0.64†	0.54 ± 0.59†	0.50 ± 0.48†	0.48 ± 0.40†	0.45 ± 0.34†	0.46 ± 0.34$^{\#\dagger}$	Condition x Set, ***P* = 0.035**; η2p = 0.215	Time, *P* = 0.067; η2p = 0.326
**HCLF**	**Pre**	0.14 ± 0.06	0.11 ± 0.03	0.12 ± 0.04	0.12 ± 0.04	0.13 ± 0.04	0.13 ± 0.04	0.14 ± 0.05	Time x Set, ***P* = 0.033**; η2p = 0.217	Set, ***P* = 0.016**; η2p = 0.243
	**Post**	0.12 ± 0.06	0.10 ± 0.00	0.11 ± 0.03	0.10 ± 0.00	0.10 ± 0.00	0.12 ± 0.04	0.10 ± 0.00$^{\#}$	Condition x Time x Set, *P* = 0.159; η2p = 0.153	
		**Blood Glucose (mg/dL)**								
**LCHF**	**Pre**	90.3 ± 14.6	89.5 ± 11.3	98.5 ±17.5	100.0 ± 23.0	103.4 ± 27.2	103.6 ± 24.8	106.0 ± 26.0	Condition x Time, *P* = 0.126; η2p = 0.240	Condition, ***P* = 0.030**; η2p = 0.426
	**Post**	80.2 ± 9.8*	82.6 ± 10.6*	88.8 ± 14.2*	90.3 ± 17.3*	88.7 ± 15.6*	89.5 ± 12.2*	96.4 ± 16.0*	Condition x Set, *P* = 0.826; η2p = 0.050	Time, *P* = 0.272; η2p = 0.132
**HCLF**	**Pre**	94.9 ± 15.7	93.9 ± 19.1	95.8 ± 18.9	102.7 ± 26.5	102.4 ± 24.5	108.7 ± 26.5	108.3 ± 26.7	Time x Set, *P* = 0.333; η2p = 0.115	Set, ***P* = 0.012**; η2p = 0.253
	**Post**	94.8 ± 11.1	101.0 ± 18.8	103.3 ± 17.0	104.6 ± 19.0	106.9 ± 29.5	105.3 ± 31.0	103.9 ± 31.6	Condition x Time x Set, *P* = 0.812; η2p = 0.052	
		**Blood Lactate (mmol/L)**								
**LCHF**	**Pre**	2.32 ± 3.20	4.45 ± 2.24	5.05 ± 3.38	5.13 ± 4.05	5.47 ± 3.87	5.08 ± 3.41	5.68 ± 3.29	Condition x Time, *P* = 0.752; η2p = 0.012	Condition, *P* = 0.821; η2p = 0.006
	**Post**	0.94 ± 0.41	3.82 ± 1.52	4.60 ± 2.80	5.34 ± 2.98	4.40 ± 2.94	5.27 ± 2.72	6.52 ± 2.92	Condition x Set, *P* = 0.140; η2p = 0.159	Time, *P* = 0.343; η2p = 0.100
**HCLF**	**Pre**	1.13 ± 0.71	4.82 ± 2.10	5.43 ± 2.53	5.50 ± 2.58	5.67 ± 2.50	5.49 ± 2.71	6.24 ± 2.83	Time x Set, *P* = 0.489; η2p = 0.093	Set, ***P* < 0.001**; η2p = 0.618
	**Post**	1.25 ± 0.96	4.88 ± 1.61	5.12 ± 2.18	5.24 ± 3.24	4.88 ± 2.59	4.94 ± 2.70	5.66 ± 2.69	Condition x Time x Set, *P* = 0.075; η2p = 0.185	

Finger capillary blood glucose, *R*-β-hydroxybutyrate, and lactate (*n* = 10). Values are Mean ± SD. Abbreviations: Pre-ex = immediately before repeated sprint protocol; Post-Ex = immediately after repeated sprint protocol; LCHF = low carbohydrate high fat diet; HCLF= high carbohydrate low fat diet. * = $p < 0.05$ difference between diets; $^{\#}$ = $p < 0.05$ difference for Pre vs. Post within same condition; † = $p < 0.05$ difference between LCHF and HCLF at same time point;

Table S4 Cardiometabolic Data (*n* = 10)

Variable	LCHF			HCLF			*P-Value*; η2p		
	**Pre Day -1**	**Post Day 31**	**Change Mean (95% CI)**	**Pre Day -1**	**Post Day 31**	**Change Mean (95% CI)**	**Condition**	**Time**	**Interaction**
**Average Data**									
**Insulin** (µU/ml)	6.37 ± 2.72	5.56 ± 1.17	-0.81 (-2.6, 0.9)	5.71 ± 1.73	5.88 ± 1.79	0.17 (-1.7, 2.0)	0.740; 0.013	0.626; 0.028	0.350; 0.097
**hsCRP** (mg/l)	0.75 ± 0.48	0.60 ± 0.43	-0.15 (-0.5, 0.2)	0.56 ± 0.47	0.78 ± 0.56	0.22 (-0.2, 0.6)	0.966; 0.000	0.753; 0.012	0.134; 0.232
**HbA1C** (%)	5.29 ± 0.59	5.13 ± 0.64	-0.16 (-0.4, 0.0)	4.98 ± 0.85	5.04 ± 0.53	0.06 (-0.3, 0.5)	0.088; 0.289	0.472; 0.059	0.388; 0.084
**Triglycerides** (mg/dL)	76.7 ± 35.0	70.5 ± 16.4	-6.20 (-24.9, 12.6)	83.2 ± 30.9	74.8 ± 15.8	-8.4 (-26.3, 9.5)	0.338; 0.102	0.368; 0.091	0.672; 0.021
**Total Cholesterol** (mg/dL)	217.2 ± 29.4	237.9 ± 24.1[#†]	20.7 (0.8, 40.6)	215.3 ± 30.4	208.4 ± 19.5	-6.90 (-24.3, 10.5)	**0.001**; 0.713	0.349; 0.098	**0.012**; 0.519
**HDL-C** (mg/dL)	57.4 ± 15.5	68.2 ± 12.8[†]	10.8 (1.9, 19.6)	59.3 ± 14.6	56.8 ± 13.5	-2.5 (-11.2, 6.2)	0.104; 0.267	0.212; 0.167	**0.020**; 0.472
**LDL-C** (mg/dL)	144.0 ± 23.3	154.7 ± 18.7[#]	10.7 (-8.4, 29.9)	138.6 ± 28.9	136.6 ± 19.9	-2.00 (-17.3, 13.3)	**0.030**; 0.425	0.423; 0.073	0.289; 0.124
**VLDL** (mg/dL)	15.3 ± 6.99	14.1 ± 3.28	-1.20 (-4.9, 2.5)	16.4 ± 6.11	15.0 ± 3.09	-1.40 (-5.1, 2.3)	0.392; 0.082	0.423; 0.073	0.855; 0.004

To convert to SI units, multiply total cholesterol, LDL-C and HDL-C (mg/dL) × 0.0256 = mmol/L; multiply triglycerides (mg/dL) × 0.0113 =mmol/L. Values are Mean ± SD (*n* = 10). Bold face denotes statistical significance ($p < 0.05$). LCHF, low carbohydrate high fat; HCLF, high carbohydrate low fat; TC = total cholesterol; HDL-C = high density lipoprotein cholesterol; LDL-C = low density lipoprotein cholesterol; TG = triglycerides; VLDL = very low-density lipoprotein; HbA1c = glycated hemoglobin.
[#] = significant difference from Pre to Post; † = significant interaction Post-diet ($p < 0.05$).

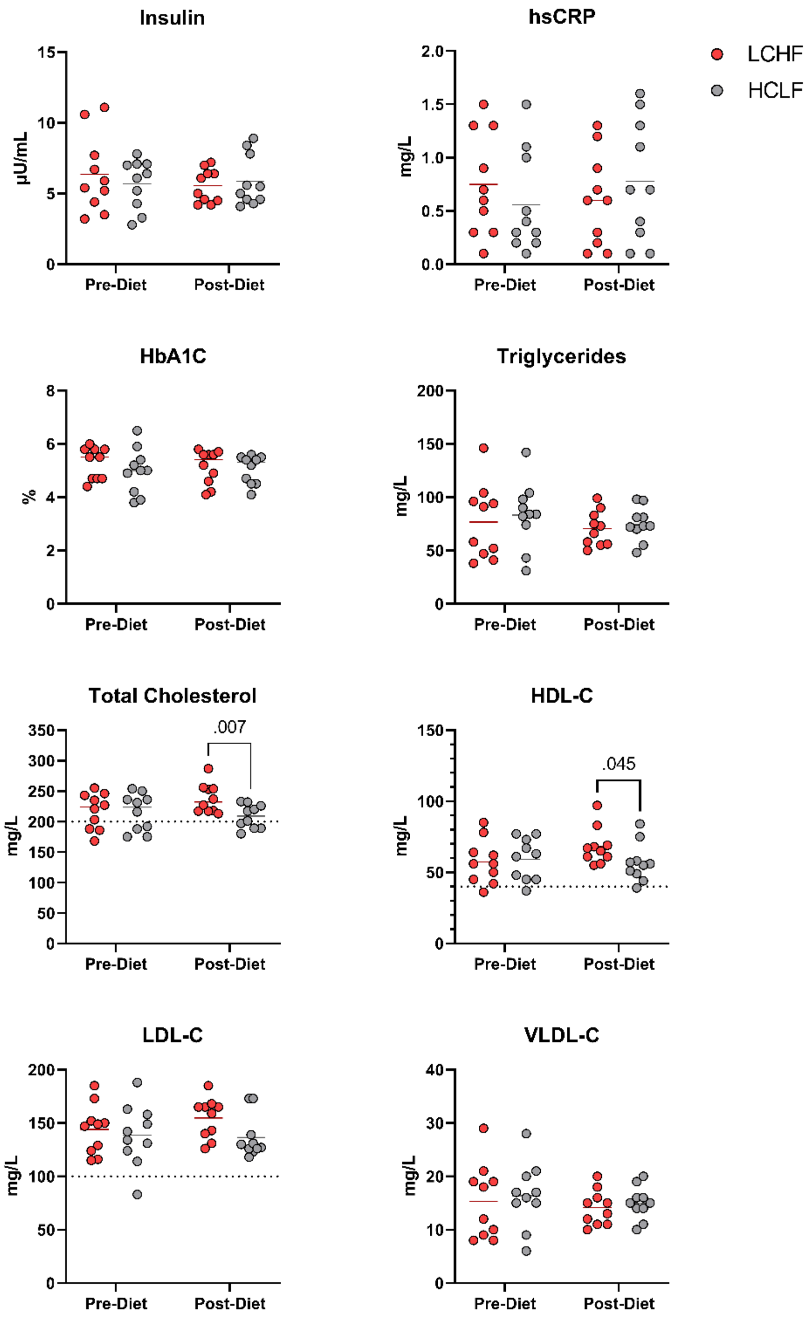

Insulin
hsCRP
LCHF
HCLF
HbA1C
Triglycerides
Total Cholesterol
.007
HDL-C
.045
LDL-C
VLDL-C
µU/mL
mg/L
%
Pre-Diet
Post-Diet

Figure S1. Individual cardiometabolic responses. *n* = 10. Horizontal bar denotes the mean. Dashed lines indicated recommended values (https://www.nhlbi.nih.gov/files/docs/public/heart/wyntk.pdf). LCHF, low carbohydrate high fat; HCLF, high carbohydrate low fat; HDL-C = high-density lipoprotein cholesterol; LDL-C = low-density lipoprotein cholesterol; VLDL-C = very low-density lipoprotein; HbA1c = glycated hemoglobin; hsCRP, high-sensitivity C-reactive protein.
